# Alkylation of Staurosporine to Derive a Kinase Probe for Fluorescence Applications

**DOI:** 10.1002/cmdc.201500589

**Published:** 2016-03-23

**Authors:** Alexander J. M. Disney, Barrie Kellam, Lodewijk V. Dekker

**Affiliations:** ^1^ School of Pharmacy, Centre for Biomolecular SciencesUniversity of NottinghamNottinghamNG7 2RDNottinghamshireUK

**Keywords:** alkylation, fluorescein, fluorescent probes, kinases, screening

## Abstract

The natural product staurosporine is a high‐affinity inhibitor of nearly all mammalian protein kinases. The labelling of staurosporine has proven effective as a means of generating protein kinase research tools. Most tools have been generated by acylation of the 4′‐methylamine of the sugar moiety of staurosporine. Herein we describe the alkylation of this group as a first step to generate a fluorescently labelled staurosporine. Following alkylation, a polyethylene glycol linker was installed, allowing subsequent attachment of fluorescein. We report that this fluorescein–staurosporine conjugate binds to cAMP‐dependent protein kinase in the nanomolar range. Furthermore, its binding can be antagonised with unmodified staurosporine as well as ATP, indicating it targets the ATP binding site in a similar fashion to native staurosporine. This reagent has potential application as a screening tool for protein kinases of interest.

## Introduction

The natural product staurosporine, first described in 1977,^1^ has been demonstrated to be a high‐affinity inhibitor of nearly all mammalian protein kinases.^2^, ^3^ Staurosporine binds to the ATP binding site of the kinase, making multiple contacts with the hinge region and the N‐ and C‐terminal lobes of the catalytic domain. Staurosporine itself is not used as a therapeutic agent, but has proven invaluable in the discovery of novel anticancer drugs based on kinase inhibition.^4^, ^5^ Furthermore, it has been modified to generate functionalised molecular tools such as immobilised staurosporine to capture kinases,^6^ a staurosporine‐based photoaffinity probe,^7^ a cell‐permeable affinity‐based probe for kinome labelling,^8^ a highly selective bivalent staurosporine‐tethered peptide ligand,^9^ and a fluorescent staurosporine conjugate.^10^

The molecular interactions between staurosporine and numerous protein kinase catalytic domains have been determined in great detail in co‐crystallisation experiments.^11^, ^12^, ^13^, ^14^, ^15^, ^16^ The main polar interaction points on staurosporine involve the lactam oxygen and nitrogen atoms, the tetrahydropyran moiety, as well as the 3′‐methoxy and 4′‐methylamine side groups (see Supporting Information for numbering). Interactions at the 4′‐methylamine contribute to binding, and the number of interactions taking place at this group appears to correlate with affinity. Both hydrogen bond and ionic interactions of this group have been implicated in binding, with different kinases engaging it in a different fashion.^17^ For example, cAMP‐dependent protein kinase (PKA) is predicted to make a hydrogen bond as well as an ionic interaction, whilst the EGF receptor kinase relies on an ionic interaction and Fyn kinase on a hydrogen bond interaction with this group.

To derivatise staurosporine, most strategies have relied upon modification of this 4′‐methylamine group, usually involving an acylation step. The resulting amide may be predicted to display decreased basicity with respect to the nitrogen atom, affecting its ability to act as hydrogen bond acceptor. As such, the acyl‐based chemical modification may result in some loss of affinity of the probe relative to staurosporine itself. This has indeed been suggested as one of the reasons why, compared with staurosporine, a probe obtained by acylation of the secondary amine showed decreased affinity to the kinase ASK1.^10^ However, acylated tools have been shown to retain kinase binding and in some cases even showed a slight increase in affinity (e.g., Ref. 8). As an alternative to the acylation modification, we have sought to derivatise staurosporine using alkylation of the 4′‐methylamine, which does not lead to formation of an amide. We report a high‐affinity fluorescent ligand, consisting of staurosporine linked to fluorescein via a polyethylene glycol linker and show that this can be used to interrogate the ATP binding site by simple fluorescence polarisation detection. We show that the ligand is useful to predict compound interactions at the ATP binding site of PKA and that the ligand is viable for the exploration of a wide range of different kinases.

## Results

### Synthesis

**Scheme 1:**
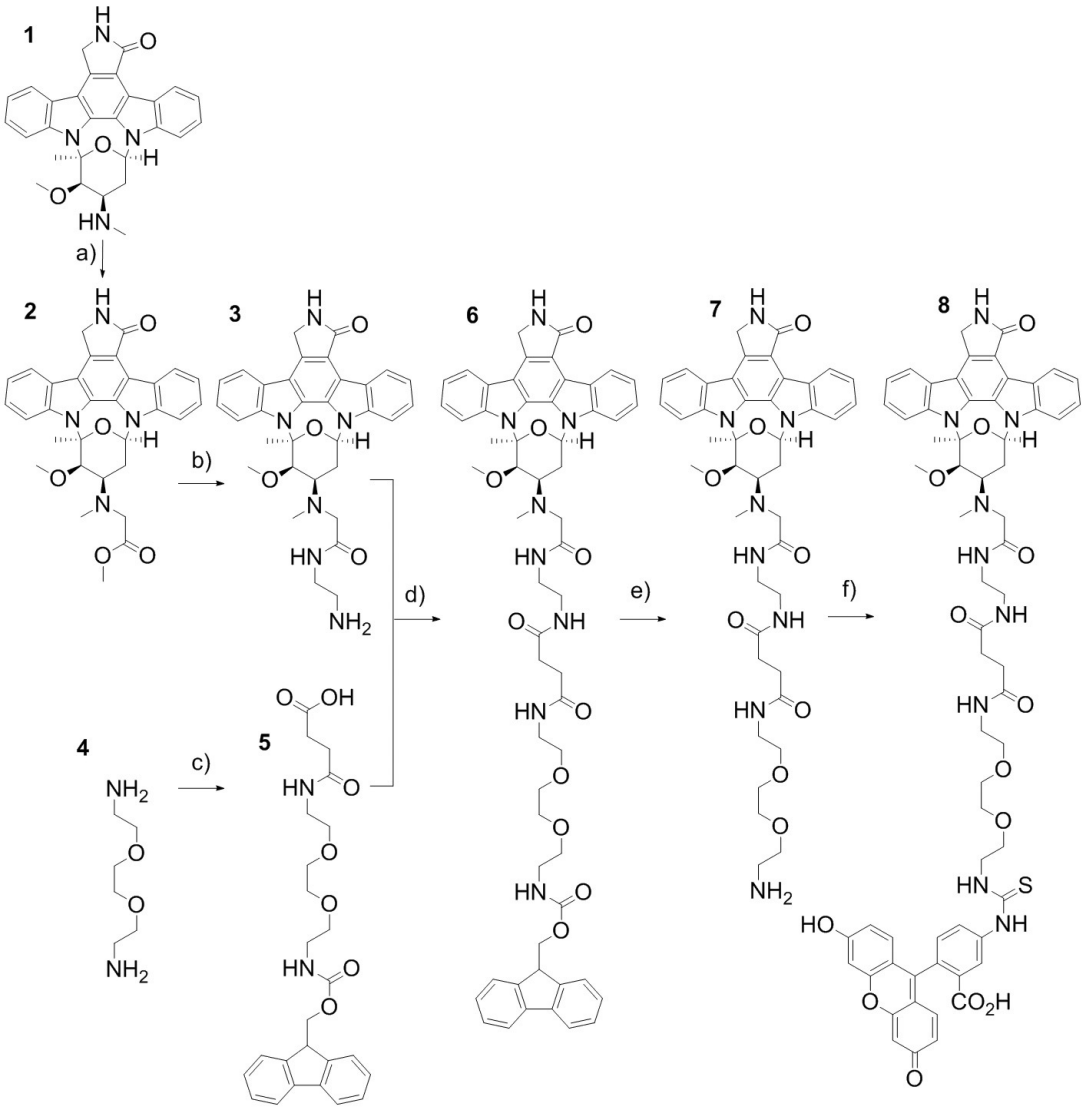
*Reagents and conditions*: a) methyl bromoacetate, potassium carbonate, THF, RT, 48 h; b) ethylenediamine, RT, 48 h; c) 1. succinic anhydride, MeCN, RT, 12 h, 2. Fmoc‐Cl, NaHCO_3_, MeCN, H_2_O, RT, 12 h; d) HBTU, DIPEA, DMF, RT, 12 h; e) diethylamine, THF, RT, 4 h; f) fluorescein isothiocyanate, DIPEA, DMF, RT, 48 h.

Staurosporine (**1**) (Scheme 1) was 4′‐N alkylated with a variety of alkyl halides of which methyl bromoacetate was found to afford the *N*‐acetyl methyl ester **2** in quantitative yield. Compound **2** was sufficiently pure to use without further purification so was aminolysed directly using ethylenediamine to give compound **3** in quantitative yield. Compound **3** provided a suitable moiety for further derivatisation of staurosporine with dyes, linkers or potentially other functionalities.

To derivatise compound **3**, we chose one of the shortest and simplest commercially available polyethylene glycol linkers available. Shorter linker strategies were attempted, but did not yield product with viable solubility and optical properties (not shown). Compound **3** was acylated using a fluorenylmethoxycarbonyl (Fmoc)‐protected diamino‐diethylene glycol linker capped at the other amino terminus with a succinic acid group (**5**) to afford **6**. Compound **5** was originally prepared by reacting commercially available 2,2‐(ethylenedioxy)bisethylamine (**4**) with succinic anhydride and Fmoc‐Cl, with a yield of 41 %.^18^ Compound **5** was subsequently acylated to the primary amine of **3** using *O*‐(benzotriazol‐1‐yl)‐*N*,*N*,*N*′,*N*′‐tetramethyluronium hexafluorophosphate (HBTU) as the coupling agent, followed by preparative HPLC, affording **6** in a yield of 47 %.

Compound **6** was deprotected using diethylamine, releasing the free amine **7**, which was again purified by RP‐HPLC. The overall yield of 43 % was somewhat disappointing, so the final step was repeated as a two‐step one‐pot procedure. Compound **6** was deprotected and then immediately coupled with fluorescein isothiocyanate to generate compound **8** with a slightly improved yield of 52 %. The overall yield for the preparation of compound **8** from staurosporine (**1**) was 24 %. NMR analysis demonstrated that the tethering point for the linker was indeed the 4′‐methylamine.

### Optical properties

**Figure 1:**
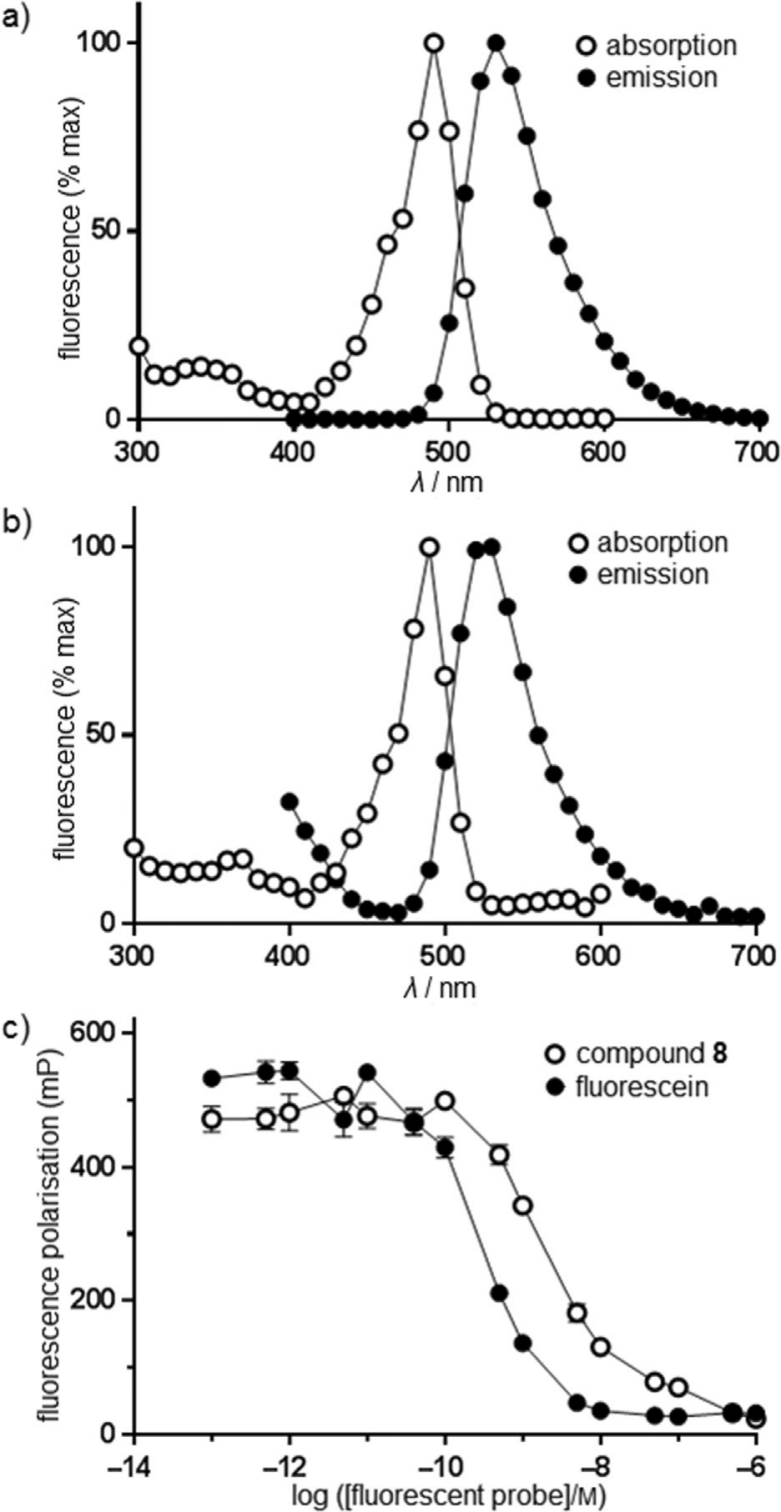
Spectral behaviour of compound **8** in comparison with fluorescein isothiocyanate. Solutions of a) fluorescein isothiocyanate and b) compound **8** were prepared at a concentration of 10 μm. Absorption and emission spectra were recorded by measuring emission at 650 nm with excitation over 300–600 nm, and by measuring emission over 400–700 nm with excitation at 350 nm, respectively. c) Fluorescence polarisation in response to fluorescein isothiocyanate and compound **8**. A set of serially diluted wells were prepared in triplicate for each of these, and fluorescence polarisation was measured as described in the Experimental Section.

Absorption and emission spectra of compound **8** and fluorescein isothiocyanate were compared. Figure 1 demonstrates that **8** displayed similar absorbance and emission profiles to those of fluorescein isothiocyanate itself. Thus the basic spectral behaviour of the fluorophore was not affected by attachment of the modified staurosporine. Next we evaluated the anisotropic properties of **8** and compared these again to those of fluorescein isothiocyanate. Fluorescein isothiocyanate provoked a full depolarisation of incident polarised light at a concentration of 10 nm (Figure 1 c). Compound **8** also depolarised the incident polarised light; however, the concentration required was higher. The minimum concentration of **8** resulting in near complete depolarisation was 50 nm (Figure 1 c). This concentration was used to measure kinase binding in subsequent assays.

### Evaluation of kinase binding

**Figure 2:**
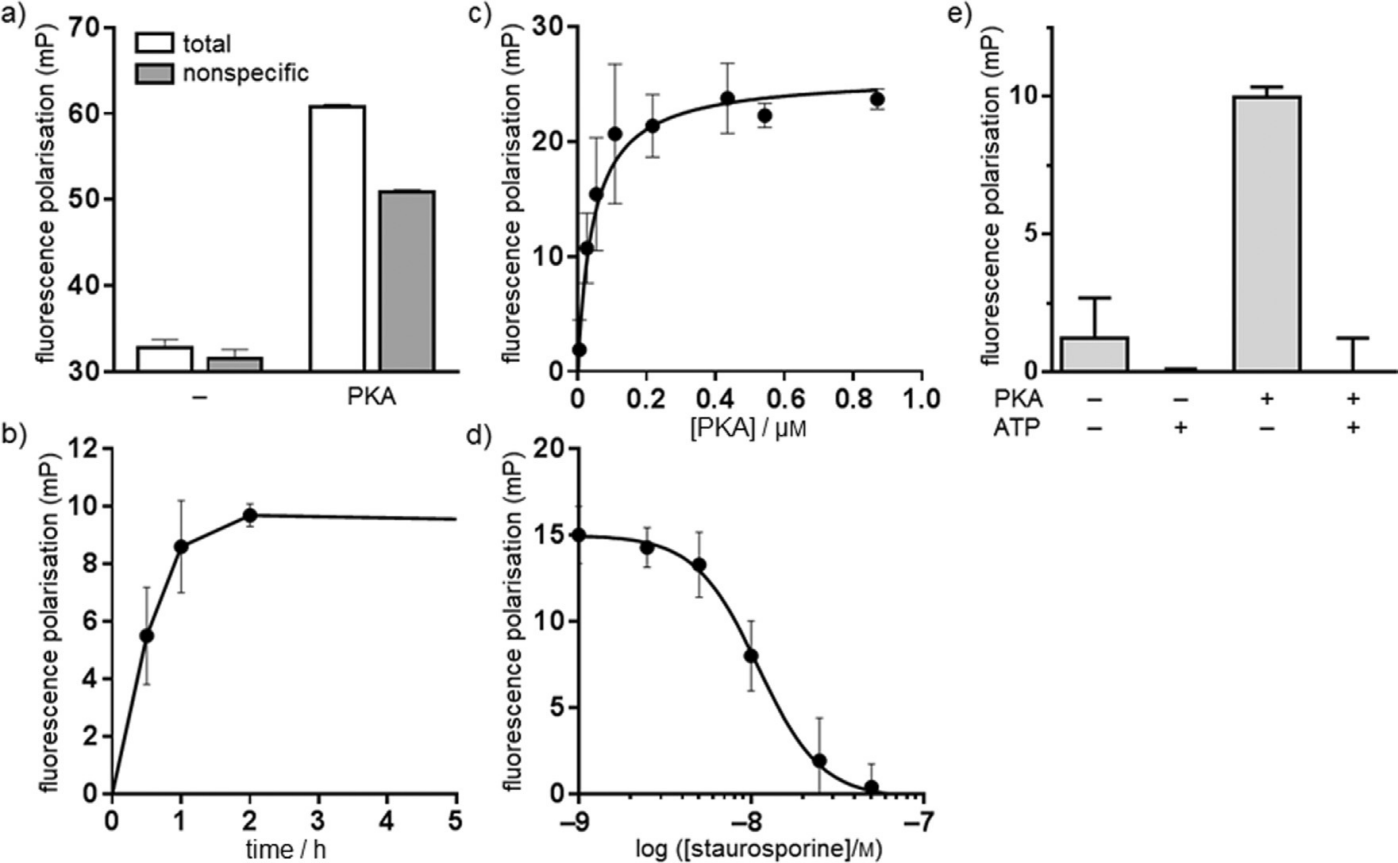
Binding of compound **8** to PKA. a) PKA (200 nm) was incubated with compound **8** (50 nm) for 120 min at 20 °C in the presence (nonspecific) or absence (total) of excess unmodified staurosporine, after which the fluorescence polarisation signal was determined (mean±SEM, *n*=2–3). b) Time dependence of specific binding of compound **8** to 200 nm PKA (mean±SEM, *n*=3). c) Concentration dependence of specific binding of PKA to 50 nm compound **8** (mean±SEM, *n*=3); nonlinear regression (one‐site binding site algorithm) was performed in Prism (version 6.04, GraphPad Software, La Jolla, CA, USA). d) Competition of binding of compound **8** (50 nm) to 200 nm PKA with unlabelled staurosporine (mean±SEM, *n*=3); nonlinear regression [log(agonist) vs. response, variable slope (four parameters)] was performed in Prism. e) Compound **8** binds to the ATP binding site of PKA: compound **8** (50 nm) was incubated with 200 nm PKA in the presence or absence of MgCl_2_ [1 mm] with ATP [1 mm], and specific binding was measured as described in panel a) (mean±SEM, *n*=2–3).

It is predicted that if compound **8** can bind to PKA, the depolarisation observed above (Figure 1 c) will be reversed. Figure 2 a shows that the fluorescence polarisation signal of 50 nm compound **8** was reversed upon incubation with a preparation of tetrameric PKA enzyme (Figure 2 a, ′total′), indicating that the kinase bound the probe. Because this PKA preparation is semi‐pure, it could be hypothesised that the reversal was not due to binding to PKA, but to nonspecific binding to non‐PKA proteins in the preparation. To assess this, a parallel incubation was carried out with excess unmodified (′cold′) staurosporine which would prevent specific binding of the probe. As expected, excess staurosporine did not affect the polarisation signal in the absence of PKA (as this is a signal derived from the probe itself). In the presence of PKA, some reversal of the polarisation signal was observed even in the presence of excess ‘cold’ staurosporine (Figure 2 a, ′nonspecific′), which we attributed to a certain level of nonspecific interaction of the probe. The difference between the total and nonspecific was then taken as specific binding to PKA. This was used throughout the study to assess probe binding. The specific binding of **8** to PKA was time dependent (Figure 2 b). Increased incubation times were associated with a decrease in the variability of the signal. To estimate the binding constant, the probe was incubated with increasing concentrations of PKA. Specific binding of compound **8** to PKA was saturable in a way that is compatible with a one‐site binding event at the kinase (Figure 2 c). The binding constant observed for binding of compound **8** was 44 nm. This is compatible with the *K*_d_ value recorded previously for unmodified staurosporine binding to PKA.^3^, ^17^, ^19^

To verify that **8** could be displaced from PKA, it was incubated at a concentration of 50 nm with PKA in the presence of increasing concentrations of unmodified staurosporine. As shown in Figure 2 d, staurosporine inhibits the binding of compound **8** to PKA in the low nanomolar range with a pIC_50_ value of 8.0±0.1 (IC_50_=11 nm). Similarly, binding of **8** to PKA was antagonised by ATP in the presence of MgCl_2_, indicating it interacted with the ATP binding site of PKA (Figure 2 e).

### Reversible binding with purified PKA catalytic subunit

**Figure 3:**
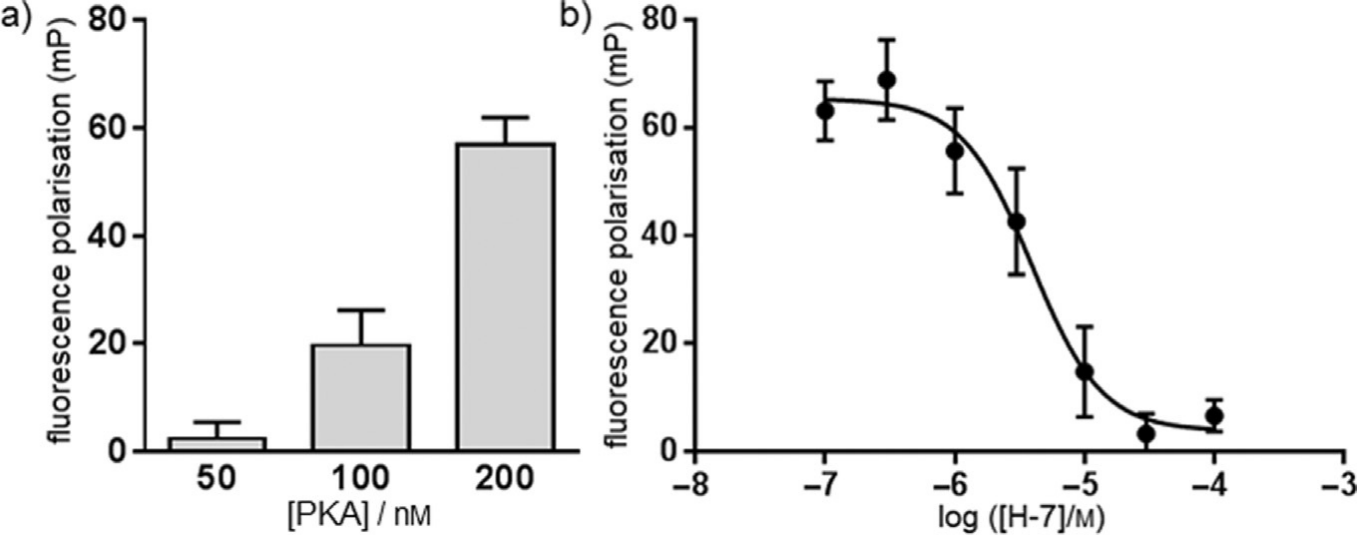
Antagonism of compound **8** binding to PKA by known kinase inhibitor H‐7. a) Binding of compound **8** to pure PKA catalytic domain. Three concentrations of the catalytic subunit from bovine PKA were incubated with 50 nm compound **8** for 120 min in the presence or absence of excess unmodified staurosporine, after which the specific fluorescence polarisation signal was determined as described in Figure 2 a (mean±SEM, *n*=2–3). b) Compound **8** (50 nm) was incubated with 200 nm PKA in the presence of increasing concentrations of H‐7, and specific binding was measured as described in Figure 2 a (mean±SEM, *n*=6). Data were fitted in GraphPad Prism (version 6.04) [log(agonist) vs. response, variable slope (four parameters)].

The above‐mentioned experiments were conducted using a semi‐pure preparation which used the whole PKA tetramer of two catalytic subunits and two regulatory subunits bound together. The binding characteristics of compound **8** indicate binding to a bona fide ATP binding site. However, as shown in Figure 2 a, a degree of nonspecific binding decreased the antagonisable window. We measured the binding of compound **8** to a purer PKA catalytic subunit preparation. The binding window for specific binding (as assessed using the procedure in Figure 2 a) increased from 10–20 mP units using semi‐pure PKA tetramer to ∼50 mP units for the purer PKA catalytic subunit (Figure 3 a).

The above data indicate that compound **8** can be used to identify compounds that bind to the ATP binding site of PKA. To confirm this further, H‐7, a known ATP binding site blocker unrelated to staurosporine was tested in the fluorescence polarisation binding assay described in Figure 3 a. As shown in Figure 3 b, H‐7 antagonised the binding of compound **8** to PKA in a concentration‐dependent fashion, with an IC_50_ value of 3.9 μm. This value is similar to the *K*_i_ reported for antagonism of PKA enzyme activity.^20^

### Interaction of compound 8 with other kinases

Whilst the experiments above indicate that compound **8** is a useful probe to identify binding of compounds to the ATP binding site of PKA, the promiscuous nature of staurosporine from which compound **8** was derived suggests that compound **8** could be a more universal kinase probe. Using the ability of the staurosporine moiety of compound **8** to inhibit kinase activity, we tested this notion by evaluating the inhibition of a range of kinases by compound **8** and comparing this with PKA inhibition.

Table 1 shows the inhibition of 50 kinases (including PKA) by compound **8** at both 100 nm and 1 μm. It can be observed that compound **8** at a concentration of 100 nm inhibited PKA by about 60 %. Given that compound **8** is a good fluorescence polarisation probe for PKA, this suggests that a kinase that is inhibited by compound **8** to a similar extent could be detected by compound **8** using fluorescence polarisation.

**Table 1:** Inhibitory activity of compound **8** against a range of serine/threonine and tyrosine kinases.^[a]^

Kinase	[**8**]/m			Kinase	[**8**]/m	
	10^−6^	10^−7^			10^−6^	10^−7^
MKK1	44±1	64±2		CK2	117±1	115±10
JNK1	83±3	82±4		DYRK1A	10±1	55±12
p38a MAPK	121±6	98±11		NEK6	82±35	84±12
RSK1	3±1	6±3		TBK1	3±2	7±2
PDK1	4±1	24±8		PIM1	20±1	70±3
PKBα	42±1	75±2		SRPK1	25±1	85±4
SGK1	34±1	77±9		EF2K	97±12	94±1
S6K1	5±1	59±16		HIPK2	74±2	93±1
PKA	8±0	40±2		PAK4	12±6	40±15
ROCK 2	4±0	54±3		MST2	4±1	10±4
PRK2	35±19	98±16		MLK3	13±1	23±5
PKCα	8±1	40±0		TAK1	4±1	53±9
PKD1	16±1	59±6		IRAK4	13±0	37±3
MSK1	6±1	49±13		RIPK2	90±2	93±9
CAMKKb	6±0	60±2		TTK	18±0	54±5
CAMK1	18±2	87±6		Src	2±1	20±14
SmMLCK	49±2	91±7		Lck	13±2	40±1
CHK2	20±0	56±5		BTK	13±2	47±1
GSK3β	11±0	47±11		JAK2	3±0	4±0
PLK1	101±1	101±4		SYK	26±5	49±9
Aurora B	11±1	24±2		EPHA2	91±8	101±5
LKB1	101±1	116±2		HER4	88±8	92±16
AMPK	1±0	1±0		IGF‐1R	56±5	116±5
MARK3	1±0	13±11		TrkA	4±0	6±1
CK1δ	118±10	109±2		VEGFR	7±1	25±4

[a] Compound **8** was tested at 10^−6^ and 10^−7^ m, and values are the percent kinase activity remaining. Data are the average of duplicate observations, with error bars indicating the spread between the duplicates. A repeat experiment was conducted with the same results.

Kinases that were inhibited by compound **8** to a similar extent as PKA include RSK1, PDK1, ROCK 2, PKCα, MSK1, GSK3β, Aurora B, AMPK (hum), MARK3, TBK1, PAK4, MST2, MLK3, IRAK4, Src, Lck, BTK, JAK2, TrkA, and VEG‐FR. Therefore, compound **8** would be predicted to be suitable to assay these kinases by fluorescence polarisation. Kinases that were clearly not inhibited by compound **8** at the concentrations tested include JNK1, p38a MAPK, PLK1, LKB1, CK1δ, CK2, EF2K, NEK6, HIPK2, EPH‐A2, and HER4; therefore, this probe would not appear to be suitable to assay these kinases. Some of these kinases (p38a MAPK, CK1δ, NEK6, CK2, EPH‐A2, EF2K and to a large extent JNK1) are, in fact, not sensitive to staurosporine itself (not shown), and for others it appears that the modification has led to a loss of binding. A number of kinases appeared to be inhibited by compound **8** but to a lesser extent than PKA, and for these kinases it would need to be established individually whether compound **8** would make a viable tool to interrogate the ATP binding site using fluorescence polarisation.

## Discussion

A fluorescein‐labelled staurosporine derivative (**8**) has been prepared by alkylation in five steps with an overall yield of 24 %. Fluorescein is widely used as fluorophore in fluorescence polarisation assays. The coupling of staurosporine to the fluorophore did not affect the basic fluorescent characteristics of the fluorophore, and the resulting probe was capable of depolarising plane‐polarised light, indicating its potential application in fluorescence polarisation assays. Using fluorescence polarisation, compound **8** was shown to bind to PKA in the nanomolar range. Compound **8** binding was successfully antagonised using unmodified staurosporine as well as ATP and H‐7, indicating it interacted with the ATP binding site of the kinase and could monitor inhibitor binding.

The most frequently employed method for attachment to staurosporine has been acylation of the 4′‐methylamine. Although the 4′‐methylamine is oriented towards solvent‐accessible space, it is involved in binding to the protein. In many crystal structures the 4′‐methylamine can be seen to be binding to acidic side chain residues in the kinase; for example, Glu127 and Glu170 in the case of PKA,^21^ and Asp807 in the case of ASK1.^22^ For kinases that hydrogen bond via aspartate or glutamate to the 4′‐methylamine nitrogen of staurosporine, a 7‐ to 80‐fold decrease in affinity was observed upon acylation of this moiety to introduce a PEG linker.^9^ However, acylation did not affect the inhibition of c‐Src by a recently developed covalently binding affinity probe.^8^ The role of the 4′‐methylamine and tetrahydropyran functionality of staurosporine may be inferred when comparing the IC_50_ values for staurosporine and K252c (staurosporine aglycone) which for PKC were 9 and 680 nm, respectively.^23^ A similar but larger difference was observed for PKA (40 and >10 000 nm).^23^ This difference in IC_50_ suggests the importance of the tetrahydropyran functionality that bears the 4′‐methylamine with respect to retaining its bonding interactions with the target protein. The tetrahydropyran ring of staurosporine is documented as being flexible, and conformational changes have been observed which include movement of the 4′‐methylamine.^24^ This flexibility of the tetrahydropyran may be partly responsible for the broad‐ranging affinity of staurosporine.

Acylation of the amine introduces an electron‐withdrawing carbonyl group that greatly decreases the basicity of the nitrogen. In alkylating rather than acylating the 4′‐methylamine, the basicity of the nitrogen is predicted to be preserved, while also maintaining the nitrogen atom's ability to act as a hydrogen bond acceptor.^25^ In the work presented herein, alkylation of the 4′‐methylamine has successfully generated a functional probe that has retained high affinity. As compound **8** has been demonstrated to bind reversibly to the ATP binding site of PKA, it could be employed to demonstrate whether a known inhibitor binds in a competitive or allosteric manner, as only competitive blockers are expected to displace compound **8**. This was confirmed using H‐7, a competitive blocker of the ATP binding site on PKA. Additionally, the fact that H‐7 displaced the probe from PKA indicates that the fluorescence polarisation format used for compound **8** could be readily used in the screening of ATP‐competitive inhibitors.

## Conclusions

Staurosporine was derivatised successfully via a five‐step alkylation route to yield a fluorescein‐based fluorescent tool. The linking and labelling strategy could be readily modified to introduce a wide range of other fluorophores or other moieties to staurosporine. The tool developed here interacted with PKA at the ATP binding site and was shown to be useful to monitor inhibitor binding at this site. We established for a large number of kinases whether or not the tool developed in this study is predicted to interact. This suggests that it is useful as probe for the identification of ATP‐competitive blockers for numerous kinases.

## Experimental Section

**General**: Solvents were obtained from Fisher and used without further purification. Reagents were obtained from Sigma–Aldrich unless otherwise stated. ^1^H and ^13^C NMR spectra were obtained with Bruker AV(I) 400 MHz, Bruker AV(III) 400 MHz, and Bruker AV(III) 500 MHz instruments. Mass spectra (ToF ES±) were recorded on a micromass LCT. Reversed‐phase HPLC was performed on a Waters 2525 gradient module coupled with a Waters 2487 dual absorbance detector set at *λ* 254 and 366 nm. A linear gradient was run over 35 min, from 100/0 phase 1 (deionised and degassed H_2_O with 0.05 % trifluoroacetic acid) to 100/0 phase 2 (90 % MeCN, 10 % H_2_O with 0.05 % trifluoroacetic acid). The analytical column used was a Phenominex Luna C_8_, 150×4.6 mm, 5 μm, at a flow rate of 1 mL min^−1^. The semipreparative column was a YMC C_8_ 150×10 mm, 5 μm, at a flow rate of 3.00 mL min^−1^. The preparative column was a Phenominex Luna C_8_, 150×30 mm, 5 μm, at a flow rate of 20 mL min^−1^. Retention times (*t*_R_) are given in minutes.

PKA was used in two forms: semi‐pure PKA from bovine heart as a whole tetramer (Sigma–Aldrich P5511), and PKA catalytic subunit purified from P5511 by column chromatography (Sigma–Aldrich P2645). PKA was added to the assay at the required number of enzyme units, and this was converted into a molar value using the specific activity of the P2645 preparation as a guide (10 units per μg protein, one unit of PKA being equivalent to 2.3 pmol of kinase).

**Fluorescence polarisation assays** were performed in 384‐well plates in a final volume of 50 μL at RT in 0.01 m phosphate buffer, 0.0027 m potassium chloride, and 0.137 m sodium chloride, pH 7.4. Fluorescence polarisation was measured on a PerkinElmer Wallac Envision 2104 Multilabel plate‐reading spectrophotometer using 485 nm excitation and 535 nm emission filters, suitable for measurement of fluorescein. Fluorescence polarisation was determined by measuring the parallel and perpendicular fluorescence emission intensity with respect to the polarised excitation light and is expressed in millipolarisation (mP) units following Equation (1):

(1) mPvalue=1000×(S-G×P)/(S+G×P)

in which *S* is the parallel and *P* is the perpendicular fluorescence signal, and *G* is the gain factor, which was set prior to the assay so that 1 nm fluorescein isothiocyanate gives a reading of 27 mP units. For fluorescence spectra in Figure 1, all solutions tested were made up in phosphate‐buffered saline (PBS) 0.01 m phosphate buffer, 0.0027 m potassium chloride, and 0.137 m sodium chloride, pH 7.4, at 25 °C with 2 % DMSO final well concentration. The wells were prepared in triplicate or quadruplicate.

**Kinase profiling** (Table 1) was performed in the Express Screen at the University of Dundee International Centre for Kinase Profiling using a radiolabel filter binding assay.^26^

**Methyl** ***N*** **‐((5*R*,7*R*,8*R*,9*S*)‐8‐methoxy‐9‐methyl‐16‐oxo‐6,7,8,9,15,16‐hexahydro‐5*H*,14*H*‐17‐oxa‐4*b*,9*a*,15‐triaza‐5,9‐methanodibenzo[*b,h*]cyclonona[*jkl*]cyclopenta[*e*]‐*as*‐indacen‐7‐yl)‐*N*‐methylglycinate (2)**. Staurosporine (LC Labs; 5 mg, 1.07×10^−5^ m) was dissolved in tetrahydrofuran (THF, 0.5 mL) and to this solution was added potassium carbonate (30 mg, 2.17×10^−4^ m, 20 equiv). Methyl bromoacetate (20 μL, 32 mg, 2.11×10^−4^ m, 20 equiv) was then added, and the mixture was stirred at room temperature (RT) for 48 h. The solvent was removed under vacuum, and the resulting solid was resuspended in THF, and the mixture was centrifuged (5 min, 8100x*g*). The supernatant was then removed under vacuum to afford a white amorphous powder (5.6 mg, 97 % yield). Purity: >99 % by HPLC, *t*_R_=16.85 min; ^1^H (400 MHz, [D_6_]DMSO, 348 K): *δ*=9.32 (1 H, d, *J*=7.7 Hz, H4), 8.24 (1 H, s, H6), 8.05–8.00 (2 H, m, H1 and H11), 7.57–7.55 (1 H, m, H8), 7.51–7.46 (2 H, m, H2 and H10), 7.37–7.27 (2 H, m, H3 and H9), 6.82 (1 H, dd, *J*=4.76, 3.15 Hz, H6′), 4.98 (2 H, s, H7), 4.34 (1 H, d, *J*=1.6 Hz, H3′), 3.71 (1 H, m, H4′), 3.56 (3 H, s, H4′′) 3.23 (2 H, m, H1′′), 2.89 (1 H, m, H5′), 2.77 (3 H, s, OCH_3_3′), 2.45 (1 H, m, H5′), 2.40 (3 H, s, NCH_3_4′), 2.18 ppm (3 H, s, CH_3_2′); ^13^C (101 MHz, [D_6_]DMSO, 293 K): *δ*=171.9, 138.3, 136.3, 132.3, 129.8, 126.3, 125.7, 125.2 125.1, 123.9, 122.6, 121.5, 120.4, 119.5, 114.7, 114.0, 113.30, 113.27, 108.9, 93.6, 81.3, 79.3, 67.0, 59.0, 53.9, 51.9, 45.4, 34.4, 30.4, 27.9 ppm; HRMS: calcd for C_31_H_30_N_4_O_5_ [*M*+H]^+^ 539.2250, found 539.2250.

***N*** **‐(2‐Aminoethyl)‐2‐(((5*R*,7*R*,8*R*,9*S*)‐8‐methoxy‐9‐methyl‐16‐oxo‐6,7,8,9,15,16‐hexahydro‐5*H*,14*H*‐17‐oxa‐4*b*,9*a*,15‐triaza‐5,9‐methanodibenzo[*b,h*]cyclonona[*jkl*]cyclopenta[e]‐*as*‐indacen‐7‐yl)(methyl)amino)acetamide (3)**. Compound **2** (5 mg, 9.29×10^−6^ m) was dissolved in ethylenediamine (1 mL, 899 mg, 1.50×10^−2^ m, 1700 equiv), and the resulting reaction mixture was stirred at RT for 48 h. Deionised water was added (2 mL), and the product was extracted into ethyl acetate, washed with deionised water (2×2 mL) and then dried using MgSO_4_ then concentrated under nitrogen. This afforded an off‐white amorphous powder (5.2 mg, 99 % yield). Purity: >99 % by HPLC, *t*_R_=12.53 min; ^1^H NMR (400 MHz, [D_6_]DMSO, 363 K): *δ*=9.34 (1 H, d, *J*=8.0 Hz, H4), 8.22 (1 H, s, H6), 8.07 (1 H, d, *J*=8.0 Hz H11), 8.02 (1 H, d, *J*=8.5 Hz, H1), 7.76 (1 H, br s, H3′′) 7.55–7.47 (3 H, m, H2, H8 and H10), 7.37 (1 H, t, *J*=7.5 Hz, H9), 7.31 (1 H, t, *J*=8.0 Hz, H3), 6.88 (1 H, dd, *J*=5.29, 3.59 Hz, H6′), 4.99 (2 H, s, H_2_7), 4.42 (1 H, d, *J*=0.3 Hz, H3′), 3.67–3.64 (1 H, m, H4′), 3.41–3.23 (4 H, m, H_2_1′’ and H_2_4′′) 3.05–2.98 (1 H, m, H5′), 2.86 (2 H, t, *J*=6.2 Hz, H_2_5′′), 2.51 (3 H, s, OCH_3_3′), 2.45 (3 H, s, NCH_3_4′), 2.41 (3 H, s, CH_3_2′) 2.40–2.33 ppm (1 H, m, H5′); ^13^C NMR (101 MHz, [D_6_]DMSO, 293 K): *δ*=172.4, 158.6, 158.4, 138.4, 136.7, 132.7, 130.3, 126.7, 126.2, 125.8, 124.3, 123.1, 122.2, 121.1, 120.0, 115.2, 114.5, 113.3, 109.6, 94.6 82.3, 59.2, 45.8, 41.1, 40.8, 38.9, 36.8, 28.1, 27.3 ppm; HRMS: calcd for C_32_H_34_N_6_O_4_ [*M*+H]^+^ 567.2675, found 567.2733.

**1‐(9*H*‐Fluoren‐9‐yl)‐3,14‐dioxo‐2,7,10‐trioxa‐4,13‐diazaheptadecan‐17‐oic acid (5)**. 2,2,‐(Ethylenedioxy)bisethylamine (1.48 mL, 10 mm) was dissolved in acetonitrile (50 mL). To this was added dropwise a solution of succinic anhydride (1.00 g, 10 mm) in acetonitrile (25 mL) over 90 min with stirring at RT.^18^ The mixture was allowed to stir for a further 90 min by which time a waxy solid had precipitated out of solution. The solvent was decanted off, and the solid was dissolved in a 1:1 mixture of acetonitrile and deionised water (100 mL). The solution was cooled in an ice bath, and to this was added dropwise a solution of Fmoc chloride (3.37 g, 13 mm, 1.3 equiv) in acetonitrile (25 mL) over 60 min. The solution was adjusted to pH 7–8 with a 5 % aqueous solution of sodium hydrogen carbonate. The mixture was left to stand overnight. The mixture was concentrated under vacuum. The solids were dissolved in 5 % sodium hydrogen carbonate (100 mL) and washed with ethyl acetate (4×75 mL). The aqueous phase was then acidified with hydrochloric acid 1 m, to between pH 2 and 3. The aqueous phase was then extracted with ethyl acetate (4×100 mL). The combined ethyl acetate extracts were washed with deionised water (2×100 mL), dried over MgSO_4_, filtered and concentrated under vacuum to afford the product as a colourless oil (1.932 g, 41.1 %). The oil was further purified by loading onto Isolute sorbent and run on silica using ethyl acetate as an eluent. This gave the light‐yellow oil that crystallised on standing (750 mg, 16.0 % yield); mp 98–100 °C. Purity: >99 % by HPLC, *t*_R_=17.37 min; ^1^H NMR (400 MHz, CDCl_3_, 293 K): *δ*=7.76 (2 H, d, *J*=7.4 Hz), 7.56 (2 H, d, *J*=7.4 Hz), 7.40 (2 H, t, *J*=7.4 Hz), 7.31 (2 H, t, *J*=7.4 Hz), 4.52–4.39 (2 H, m) 4.28–4.18 (1 H, m), 3.61 (4 H, s), 3.59–3.53 (2 H, m), 3.49–3.41 (4 H, m), 3.41–3.31 (2 H, m), 2.72–2.64 (2 H, m), 2.53–2.45 ppm (2 H, m); ^13^C NMR (101 MHz, [D_6_]DMSO, 293 K): *δ*=174.0, 171.3, 156.3, 144.0, 140.9, 127.2, 125.3, 120.2, 69.6, 65.5, 46.9, 38.7, 30.1, 29.3 ppm; HRMS: calcd for C_25_H_30_N_2_O_7_ [*M*+H]^+^ 471.2087, found 471.2151, HRMS: calcd for C_25_H_30_N_2_O_7_ [*M*−H]^−^ 469.1980, found 469.1860.

**Fluoren‐9‐ylmethyl (2‐((5*R*,7*R*,8*R*,9*S*)‐8‐methoxy‐9‐methyl‐16‐oxo‐6,7,8,9,15,16‐hexahydro‐5*H*,14H‐17‐oxa‐4b,9a,15‐triaza‐5,9‐methanodibenzo[*b,h*]cyclonona[*jkl*]cyclopenta[*e*]‐as‐indacen‐7‐yl)‐4,9,12‐trioxo‐16,19‐dioxa‐2,5,8,13‐tetraazahenicosan‐21‐yl)carbamate (6)**. Compound **3** (9.40 mg, 1.66×10^−5^ m) was dissolved in DMF (100 μL) and to this solution was added DIPEA (3.2 μL, 2 equiv). A separate solution of **5** (9.37 mg, 1.2 equiv), HBTU (7.55 mg, 1.2 equiv), and DIPEA (3.2 μL, 2 equiv) was prepared in DMF (100 μL). The two solutions were stirred for 1 min and then combined. The resulting reaction mixture was stirred for a further 12 h. The solvent was removed under vacuum and the reaction product purified by semi‐preparative RP‐HPLC to afford an amorphous white powder (8 mg, 47 % yield). Purity: >99 % by HPLC, *t*_R_=18.12 min; ^1^H NMR (500 MHz, [D_6_]DMSO, 298 K): *δ*=9.32 (1 H, d, *J*=8.0 Hz), 8.63 (1 H, s), 8.09 (1 H, d, *J*=8.0 Hz), 8.05 (1 H, d, *J*=8.5 Hz), 7.89–7.86 (4 H, m), 7.68–7.63 (2 H, m), 7.56–7.49 (3 H, m), 7.39 (3 H, t, *J*=7.1 Hz), 7.34–7.30 (3 H, m), 6.99–6.97 (1 H, m), 5.00 (2 H, s), 4.29–4.27 (2 H, m), 4.21–4.18 (1 H, m), 3.47 (4H s), 3.40–3.34 (5 H, m), 3.17 (6 H, s), 3.15–3.10 (6 H, m), 2.48 (2 H, s), 2.32–2.28 (4 H, m), 2.25 ppm (2 H, br s); ^13^C NMR (126 MHz, [D_6_]DMSO, 293 K): *δ*=206.5, 171.9, 171.8, 171.5, 156.2, 143.9, 140.7, 137.8, 136.3, 132.3, 129.7, 127.6, 127.0, 126.2, 125.8, 125.4, 125.4, 125.2, 124.9, 123.9, 122.7, 121.8, 120.8, 120.1, 119.8, 119.7, 118.1, 117.8, 115.5, 114.9, 114.2, 112.7, 109.2, 94.1, 81.6, 79.1, 69.5, 69.5, 69.1 65.3, 59.0, 54.7, 48.6, 46.7, 45.4, 40.5, 38.8, 38.5, 37.9, 30.8, 30.5, 29.1, 27.4, 26.1, 22.5, 1.2 ppm; HRMS: calcd for C_57_H_62_N_8_O_10_ [*M*+H]^+^ 1019.4622, found 1019.4639.

***N*** ^**1**^ **‐(2‐(2‐(2‐Aminoethoxy)ethoxy)ethyl)‐*N*** ^**4**^ **‐(2‐(2‐(((5*R*,7*R*,8*R*,9*S*)‐8‐methoxy‐9‐methyl‐16‐oxo‐6,7,8,9,15,16‐hexahydro‐5*H*,14*H*‐17‐oxa‐4*b*,9*a*,15‐triaza‐5,9‐methanodibenzo[*b***,***h*** **]cyclonona[*jkl*]‐cyclopenta[*e*]‐*as*‐indacen‐7‐yl)(methyl)amino)acetamido)ethyl)succinimide (7)**. Diethylamine (200 μL) was added to a stirred solution of **6** (4 mg, 3.93×10^−6^ m) in THF (800 μL) and the mixture was stirred for 4 h; completion was assessed by mass spectrometry. The solvent and excess diethylamine were removed under vacuum. The remaining solids were triturated with THF and petroleum ether and then dried under high vacuum for 6 h to afford an off‐white powder. Half of this material was then purified for analysis by semi‐preparative RP‐HPLC to afford an amorphous white powder **7** (0.7 mg, 45 % yield). Purity: >99 % by HPLC, *t*_R_=12.03 min; ^1^H NMR (400 MHz, [D_6_]DMSO/D_2_O 9:1, 293 K): *δ*=9.20 (1 H, d, *J*=8.1 Hz, H4), 8.04–7.99 (2 H, m, H1 and H11), 7.54–7.46 (3 H, m, H2, H8 and H10), 7.36 (1 H, t, *J*=7.4 Hz, H9), 7.31–7.27 (1 H, m, H3), 6.81–6.78 (1 H, m, H6′), 4.98 (2 H, s, H_2_7), 4.22 (1 H, s, H3′), 3.53 (2 H, t, *J*=5.1 Hz, CH_2_), 3.50–3.46 (4 H, m, 2CH_2_) 3.41–3.39 (1 H, m, CH), 3.35 (2 H, t, *J*=5.9 Hz, CH_2_), 3.31–3.27 (1 H, m, H4′), 3.15 (2 H, t, *J*=5.8 Hz, CH_2_), 3.06–2.81 (8 H, m, H5′, H_2_1′′, H_2_4′′, and 3CH), 2.53 (3 H, s, OCH_3_3′), 2.36 (3 H, s, NCH_3_4′), 2.33–2.28 (1 H, m, H5′), 2.27–2.21 (4 H, m, 2CH_2_), 2.00 ppm (3 H, s, CH_3_2′); ^13^C NMR (101 MHz, [D_6_]DMSO, 293 K): *δ*=139.2, 136.3, 132.3, 129.8, 128.0, 126.2, 124.9, 123.9, 122.7, 114.8, 94.2, 72.3, 69.6, 69.4, 69.0, 66.6, 60.0, 58.8, 38.6, 38.4, 34.4, 30.7, 30.5, 30.4, 21.0, 16.7 ppm; HRMS: calcd for C_42_H_52_N_8_O_8_ [*M*+H]^+^ 797.39417, found 797.3945

**2‐(6‐Hydroxy‐3‐oxo‐3*H*‐xanthen‐9‐yl)‐5‐(3‐(2‐((5*R*,7*R*,8*R*,9*S*)‐8‐methoxy‐9‐methyl‐16‐oxo‐6,7,8,9,15,16‐hexahydro‐5*H*,14*H*‐17‐oxa‐4b,9a,15‐triaza‐5,9‐methanodibenzo[*b***,***h*** **]cyclonona[*jkl*]‐cyclopenta[*e*]‐*as*‐indacen‐7‐yl)‐4,9,12‐trioxo‐16,19‐dioxa‐2,5,8,13‐tetraazahenicosan‐21‐yl)thioureido)benzoic acid (8)**. The remaining half of the deprotected material described above for the preparation of compound **7** was dissolved in DMF (100 μL) and added to a solution of fluorescein isothiocyanate (1 mg, 1.3 equiv) in DMF (100 μL). This was left to stir at RT in the dark for 12 h. Monitoring by mass spectrometry indicated unreacted precursor; therefore, further fluorescein isothiocyanate (0.76 mg, 1 equiv) was added and left to stir for a further 24 h. At this point there was little or no detectable precursor remaining, so the solvent was removed under vacuum and the material was purified by HPLC to afford an amorphous dark‐orange powder (1.2 mg, 52 % yield). Purity: >99 % by HPLC, *t*_R_‐15.60 min; ^1^H NMR (500 MHz, [D_6_]DMSO/D_2_O 9:1, 298 K): *δ*=9.25 (1 H, d, *J*=8.1 Hz), 8.26 (1 H, br s), 8.07 (1 H, d *J*=7.9 Hz), 8.02 (1 H, d, *J*=8.3 Hz), 7.70 (1 H, d, *J*=7.6 Hz), 7.52 (3 H, quintet, *J*=8.0 Hz), 7.31 (1 H, t, *J*=7.7 Hz), 7.14 (1 H, d, *J*=7.9 Hz), 6.93 (1 H, d, *J*=8.8 Hz), 6.68–6.67 (2 H, m), 6.59–6.57 (2 H, m), 6.56–6.53 (2 H, m), 4.98 (2 H, s), 4.64 (1 H, s), 3.65 (2 H, s), 3.56 (2 H, t, *J*=5.1 Hz), 3.50, (4 H, dd, *J*=4.0, 10.6 Hz), 3.34 (2 H, t, *J*=5.4 Hz), 3.13–3.11 (6 H, m), 2.84 (3 H, s), 2.45 (2 H, s) 2.36 (1 H, s) 2.27 (4 H, q, *J*=6.5 Hz), 2.19 (3 H, s), 2.07–2.06 ppm (1 H, m); HRMS: calcd for C_63_H_63_N_9_O_13_S [*M*+H]^+^ 1186.4300, found 1186.3001.

## Supporting information

As a service to our authors and readers, this journal provides supporting information supplied by the authors. Such materials are peer reviewed and may be re‐organized for online delivery, but are not copy‐edited or typeset. Technical support issues arising from supporting information (other than missing files) should be addressed to the authors.

Supplementary
