# Supplementary material for: Alkylation of Staurosporine to Derive a Kinase Probe for Fluorescence Applications
Source: ChemMedChem. 2016 Mar 23;11(9):972–9. doi: 10.1002/cmdc.201500589 (PMC4949516; doi:10.1002/cmdc.201500589)
Supplement: Supplementary file 1 — Supplementary [file CMDC-11-972-s001.pdf]

CHEMMEDCHEM Chemistry & Drug Discovery

Supporting Information

Alkylation of Staurosporine to Derive a Kinase Probe for Fluorescence Applications

Alexander J. M. Disney, Barrie Kellam, and Lodewijk V. Dekker*[a]

cmdc_201500589_sm_miscellaneous_information.pdf

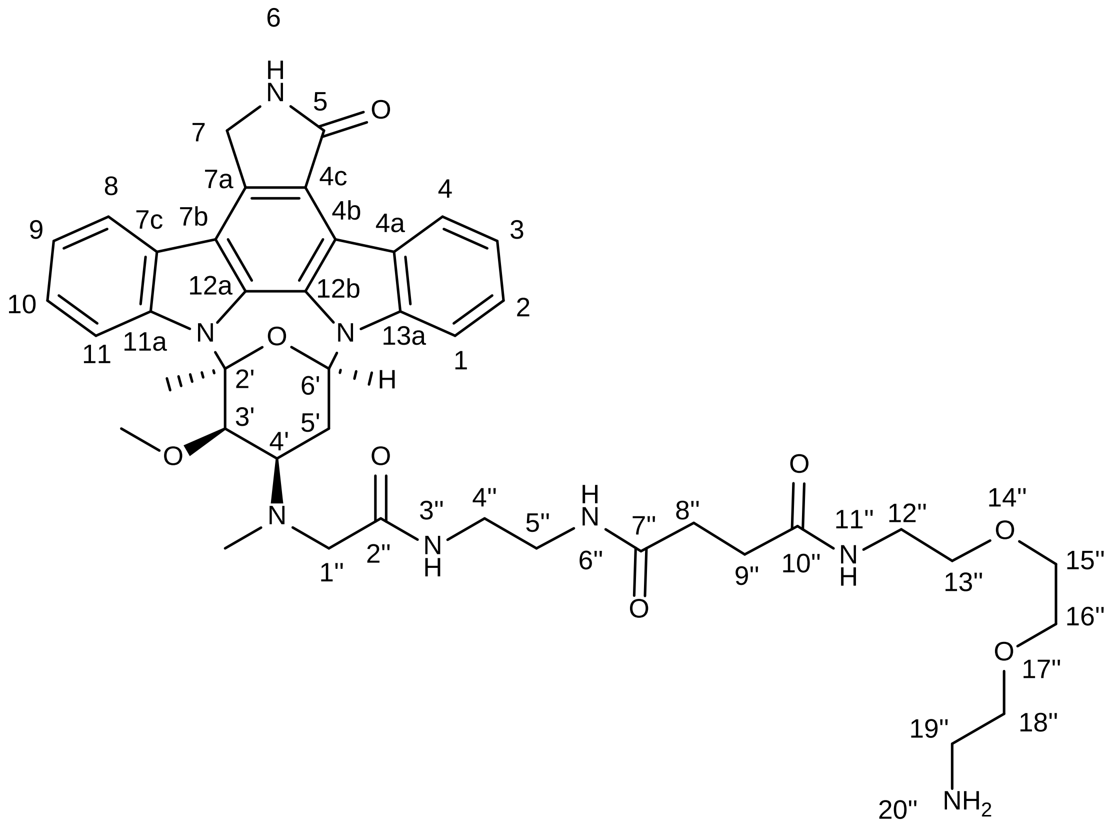

Supplement Figure: Compound **7** numbering.

^{1}H spectrum for compound **2**, DMSO D_6, 348K

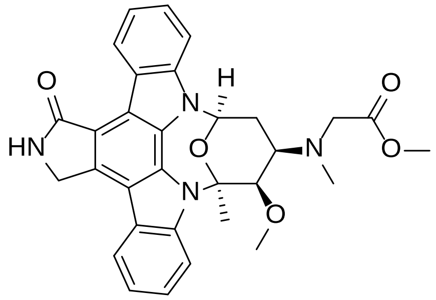

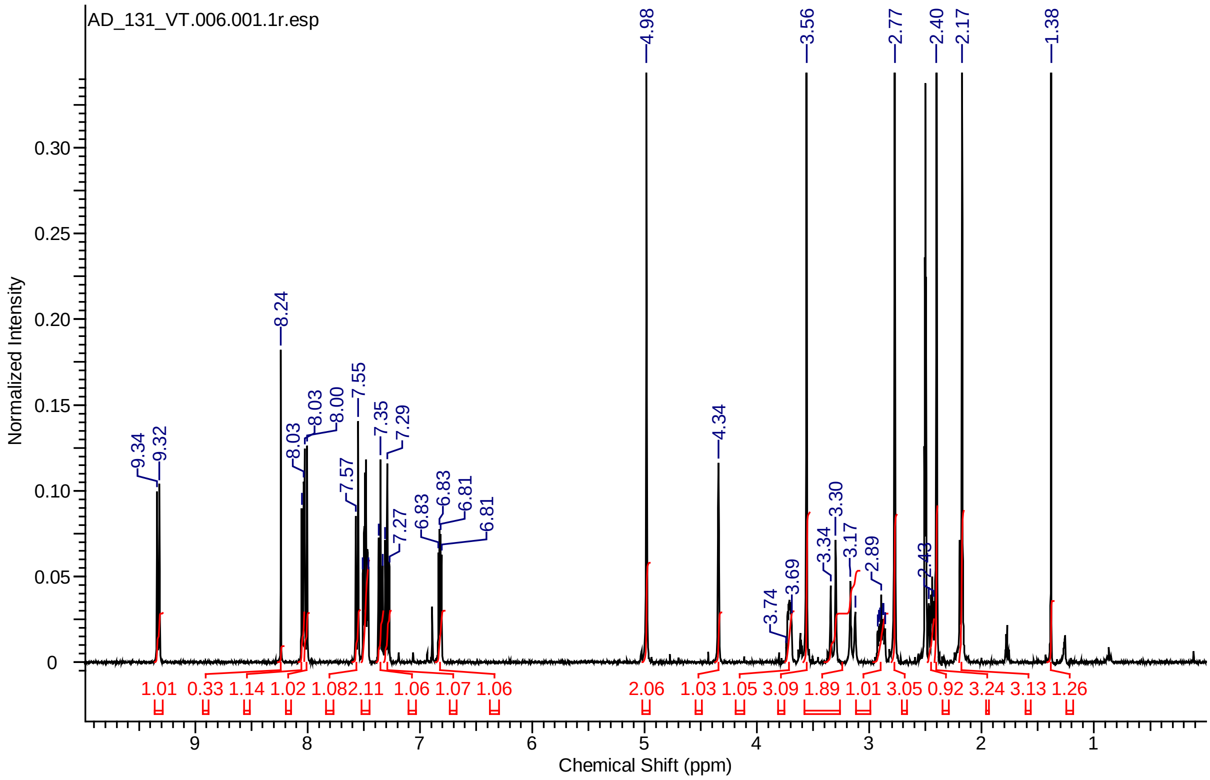

^{13}C spectrum for compound **2**, DMSO D_6, 293K

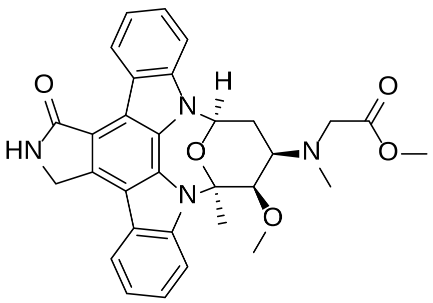

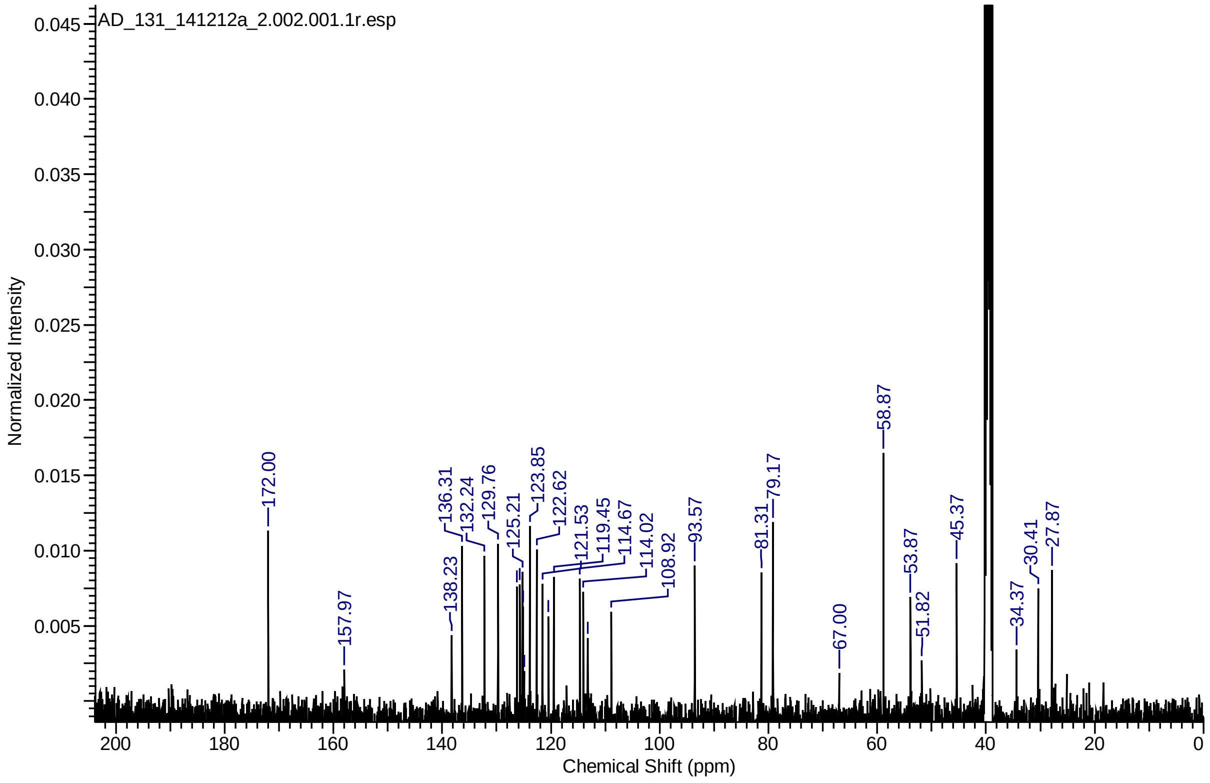

HPLC analysis of compound **2**

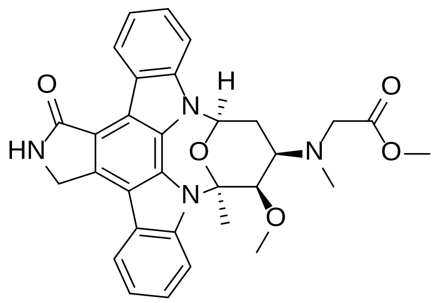

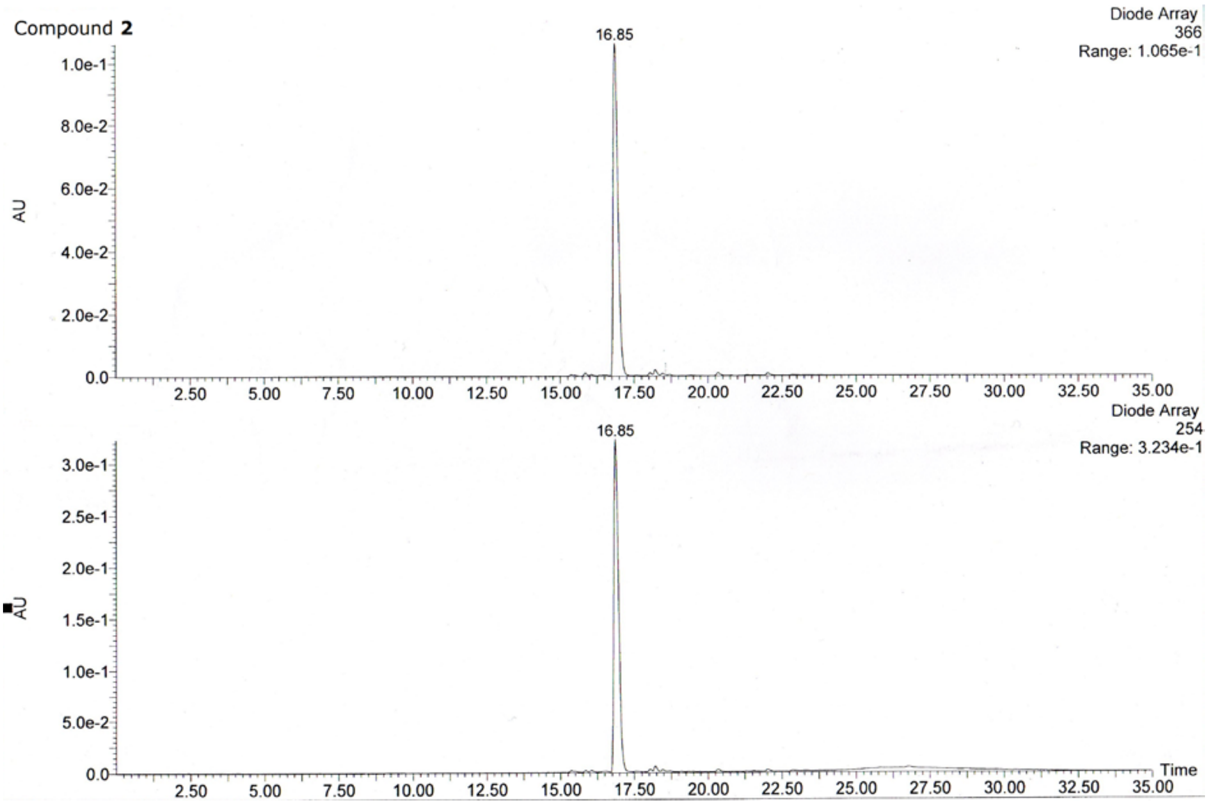

^{1}H spectrum for compound **3**, DMSO D_6, 363K

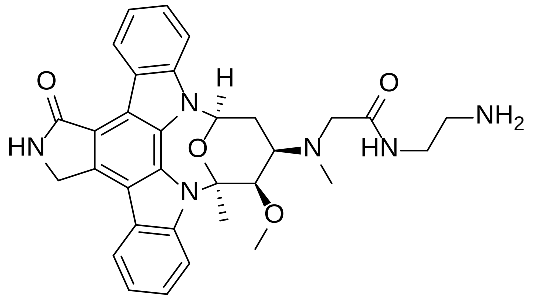

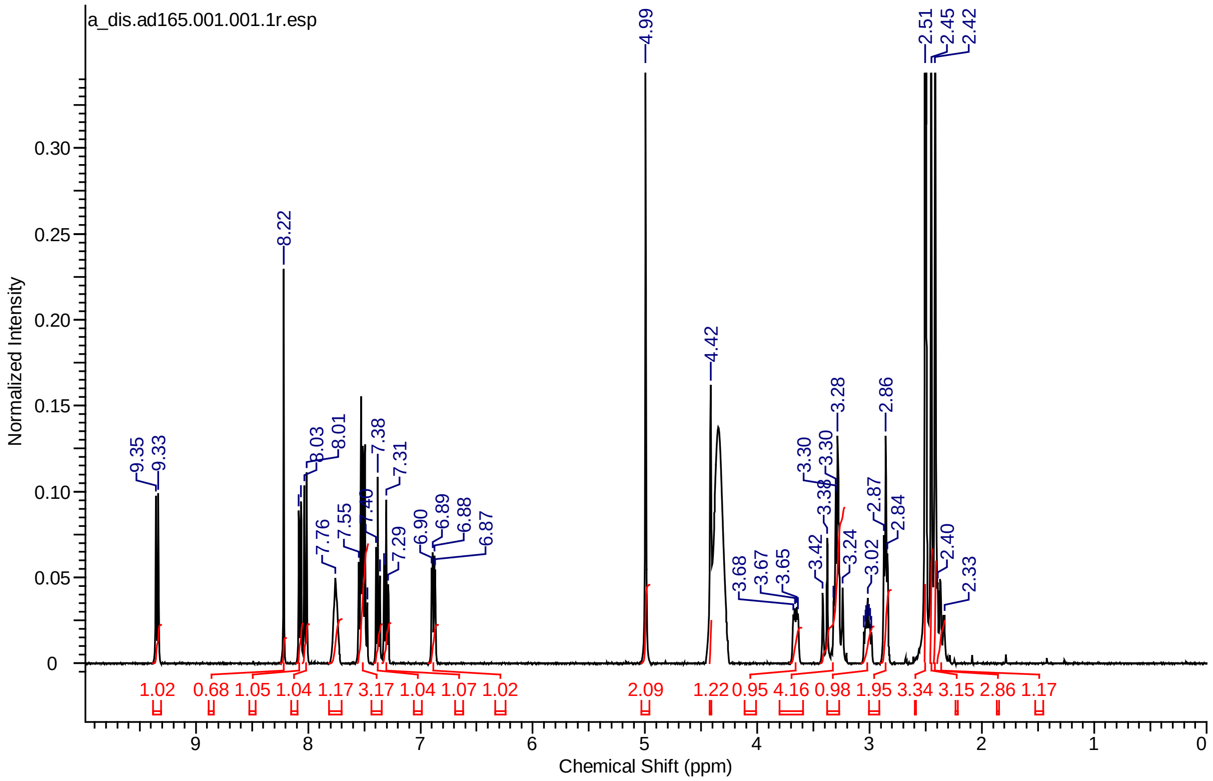

^{13}C spectrum for compound **3**, DMSO D_6, 293K

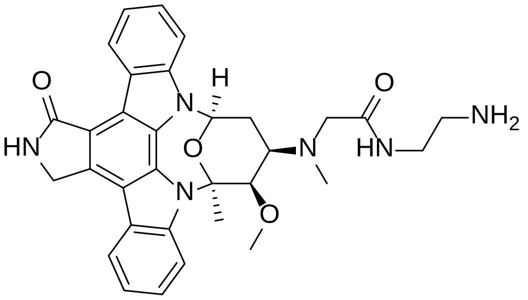

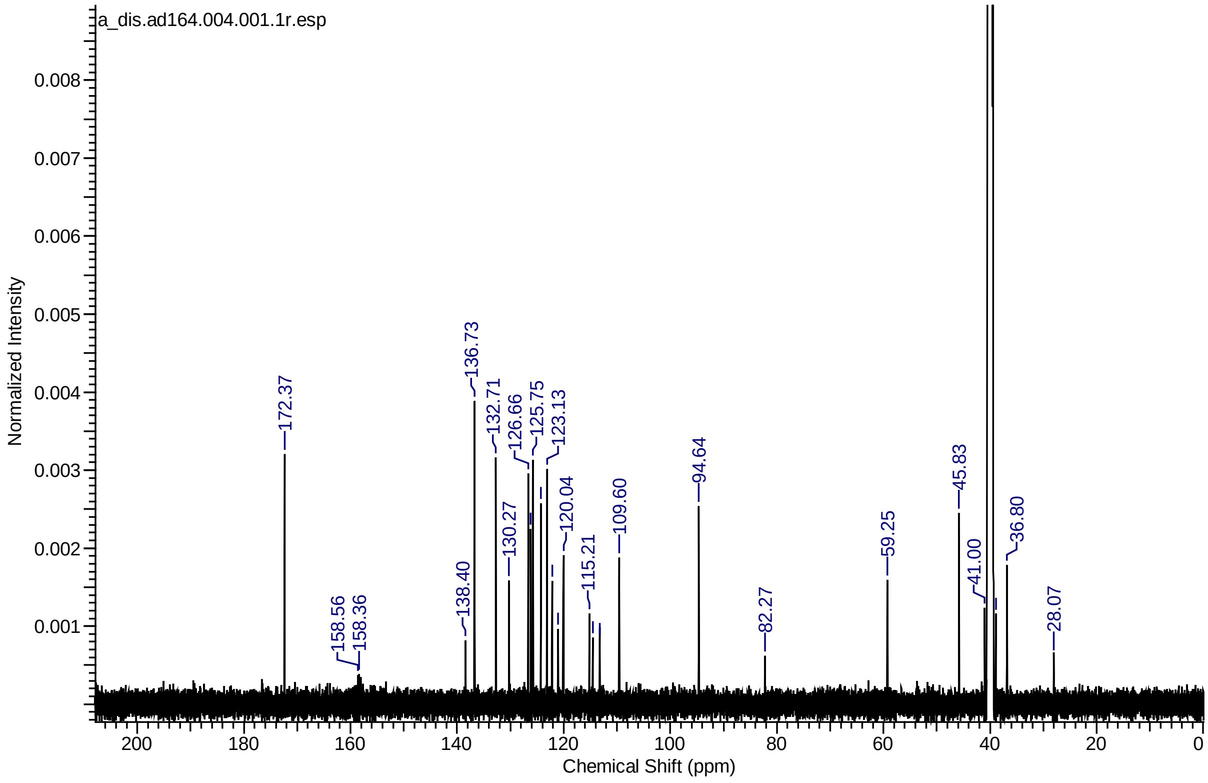

HPLC analysis of compound **3**

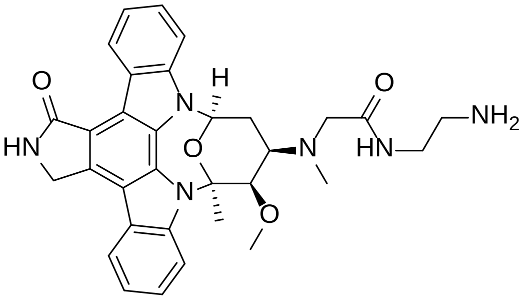

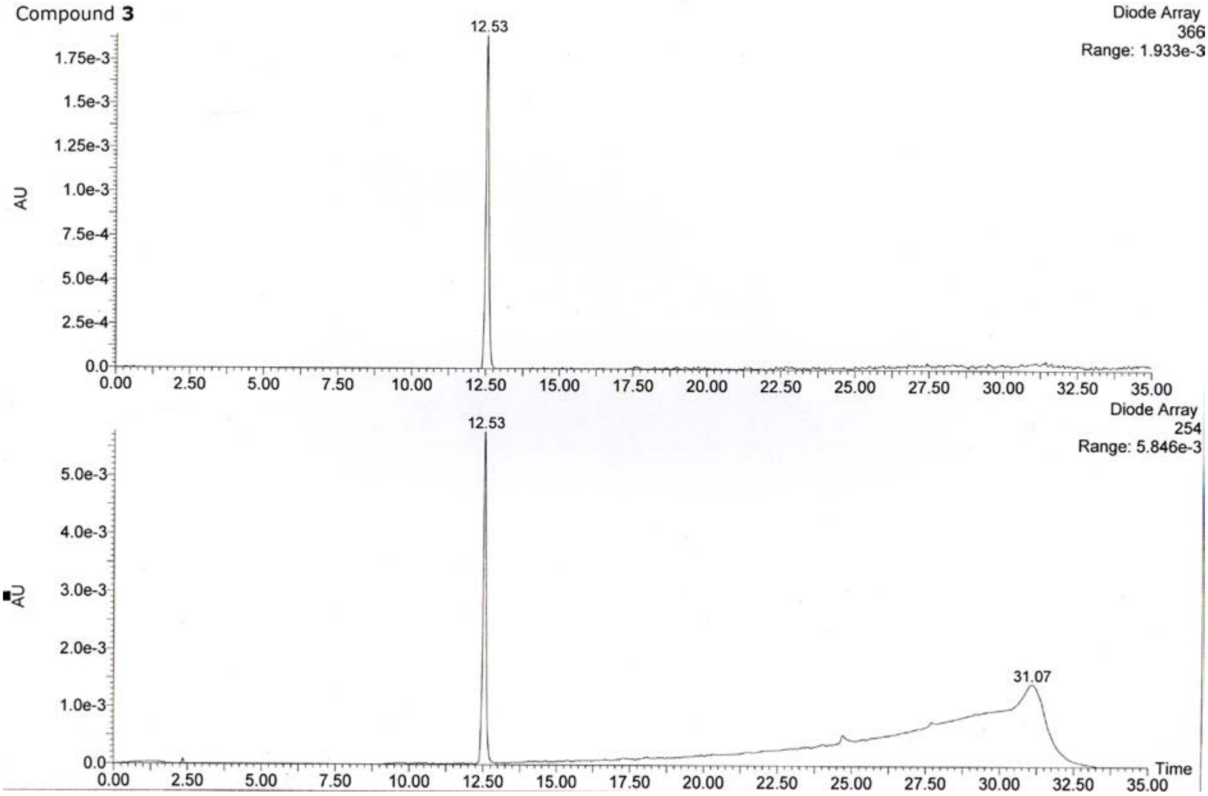

^{1}H spectrum for compound **5**, DMSO D_6 + 5% D_2O, 293K

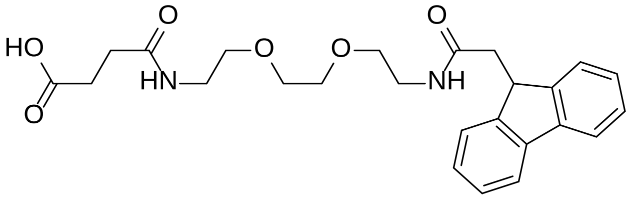

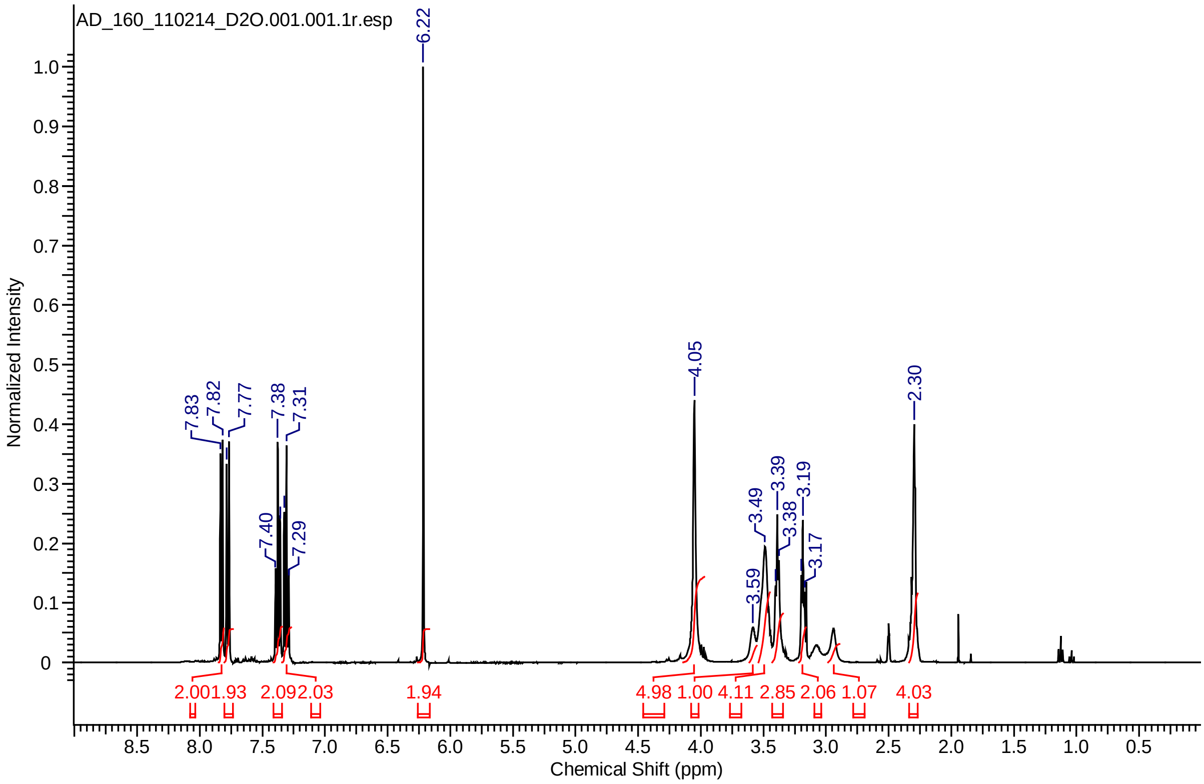

^{13}C spectrum for compound **5**, $CDCl_3$, 293K

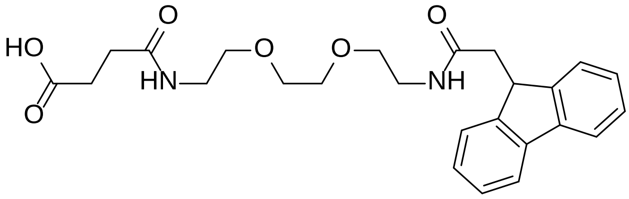

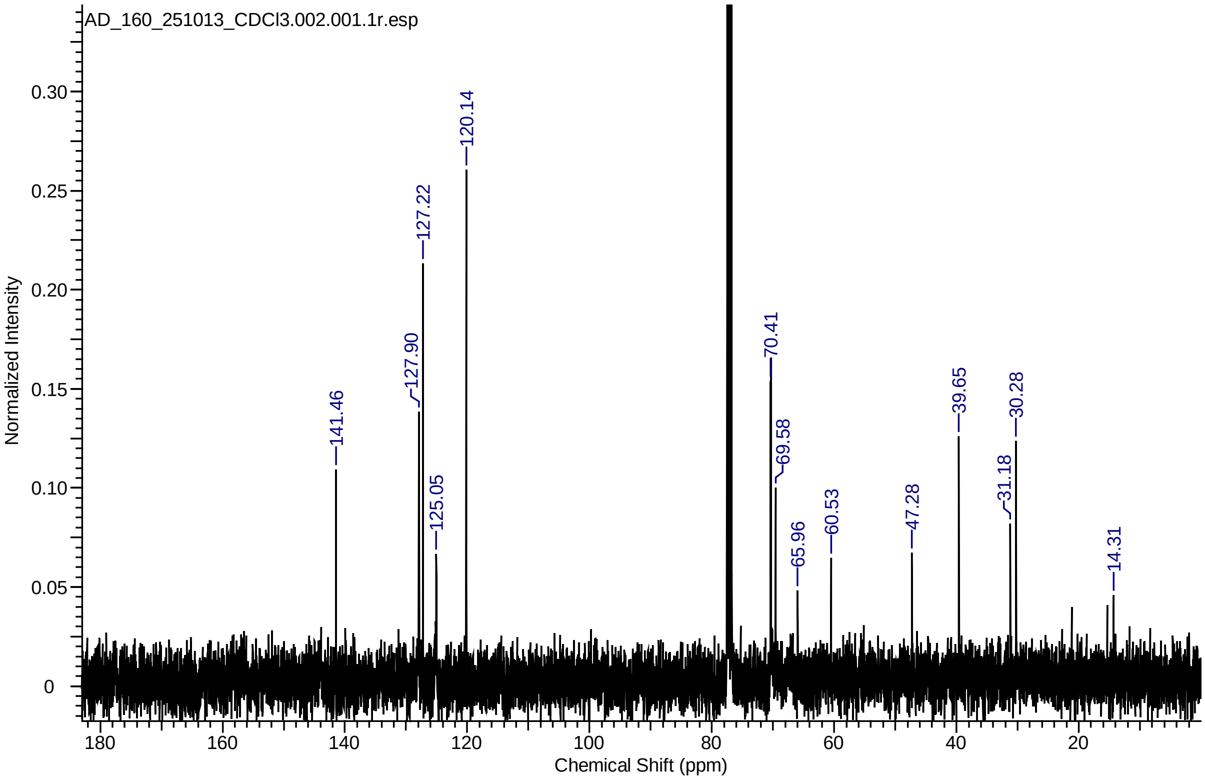

HPLC analysis of compound **5**

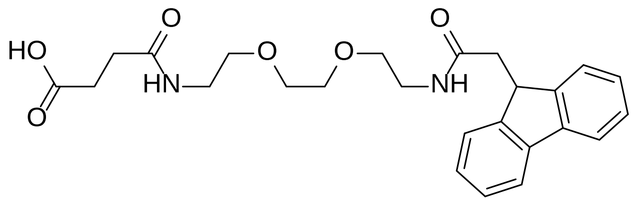

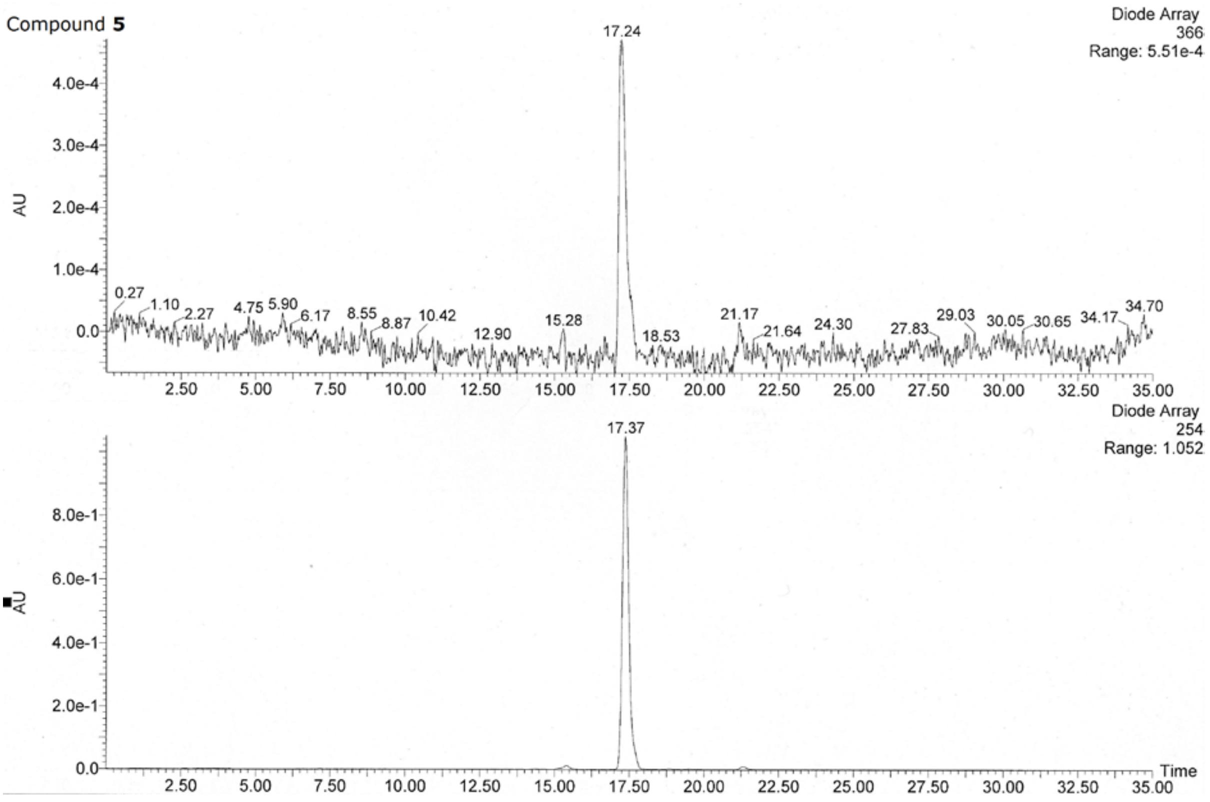

^{1}H spectrum for compound **6**, DMSO D_6, 298K

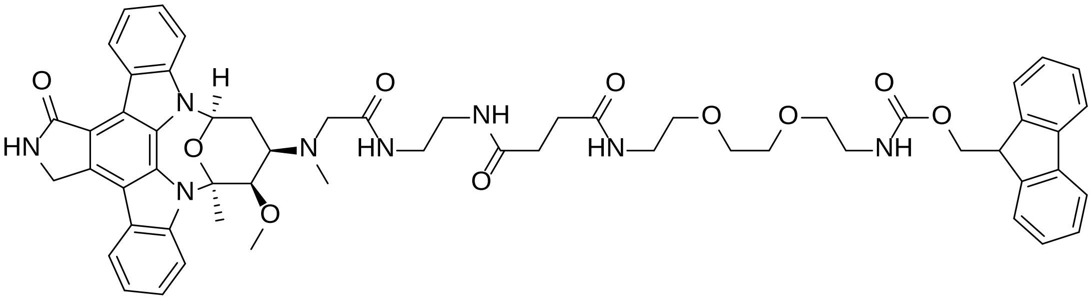

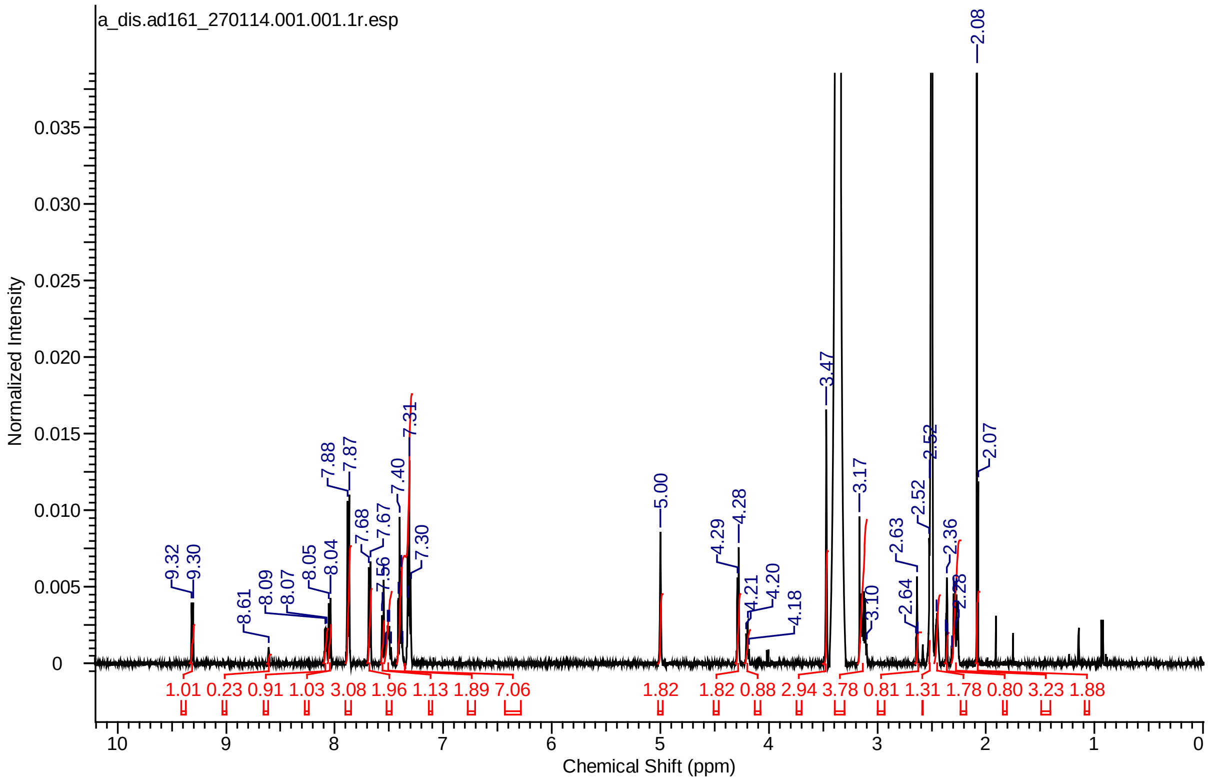

^{13}C spectrum for compound **6**, DMSO D_6, 298K

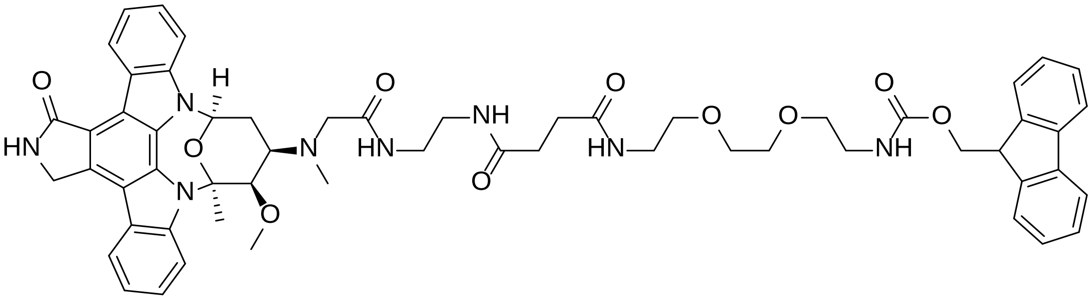

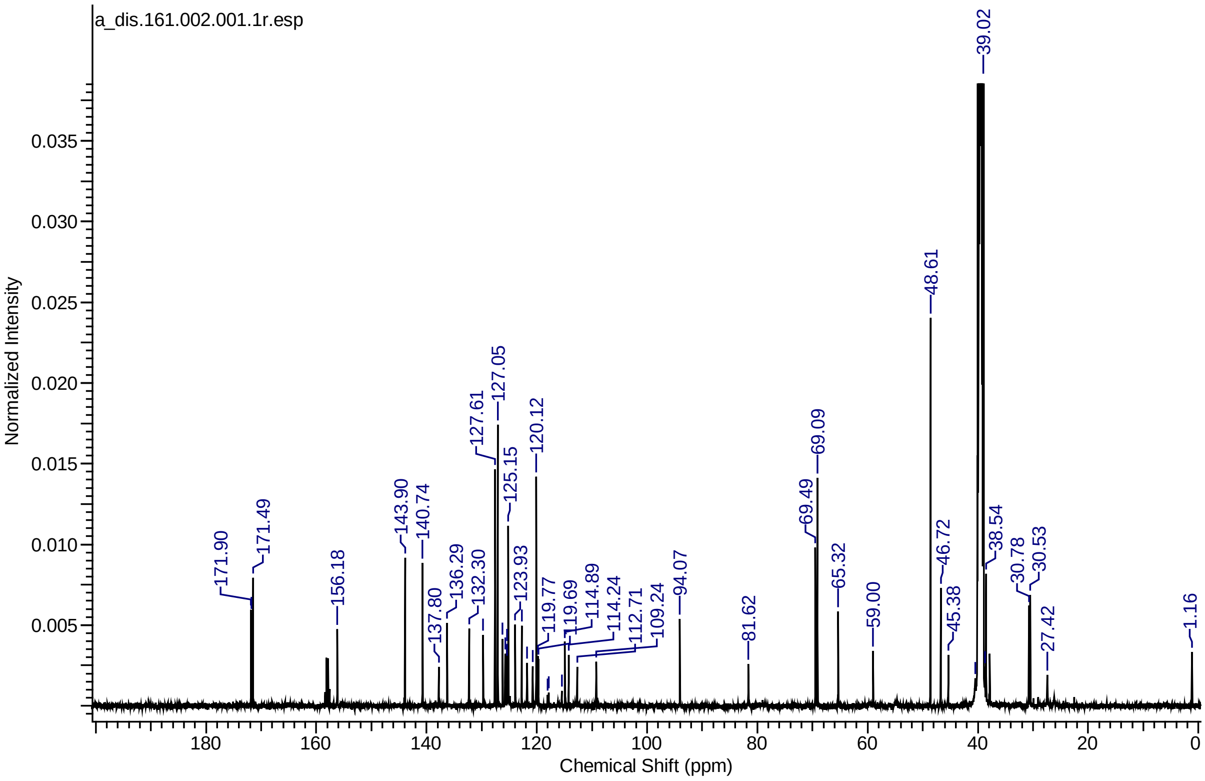

HPLC analysis of compound **6**

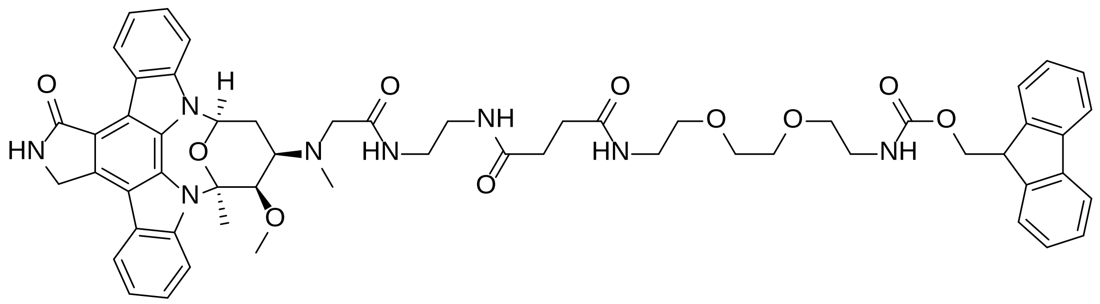

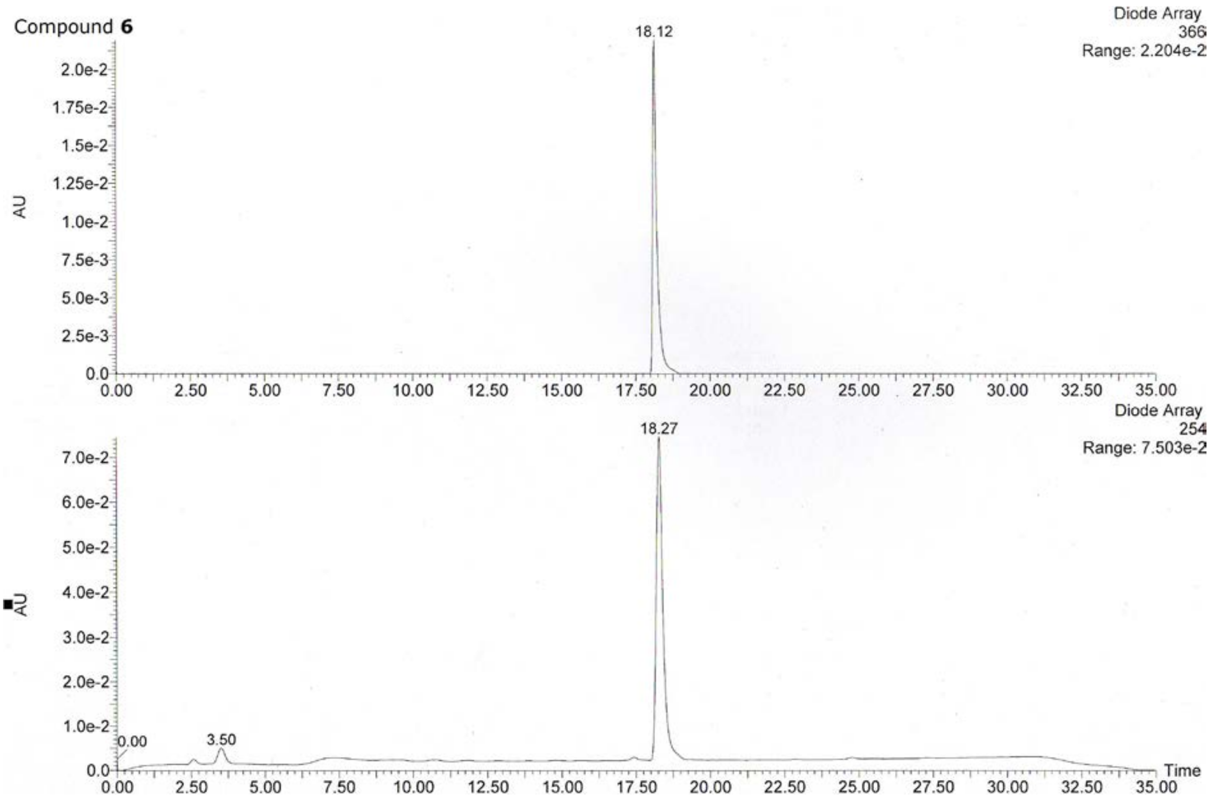

^{1}H spectrum for compound **7**, DMSO D_6 + 5% D_2O, 293K

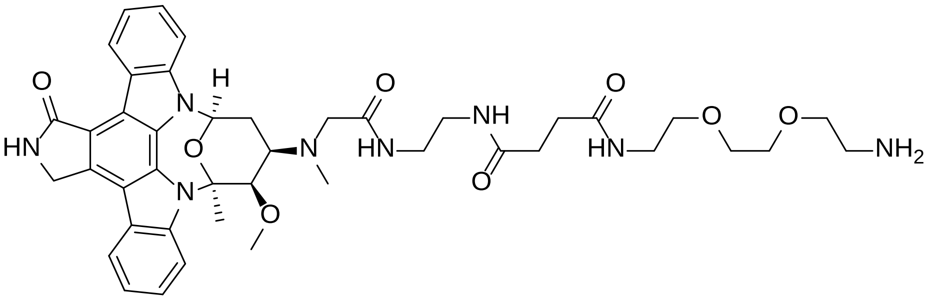

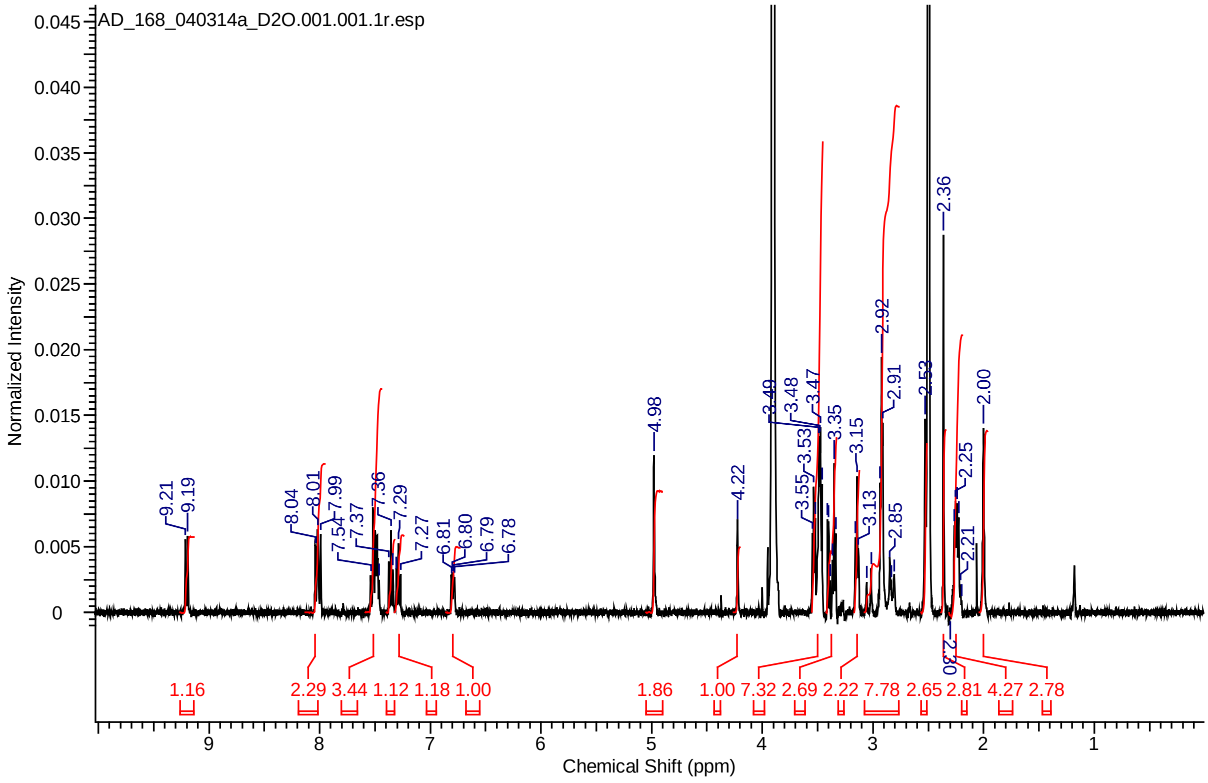

^{13}C spectrum for compound **7**, DMSO D_6, 293K

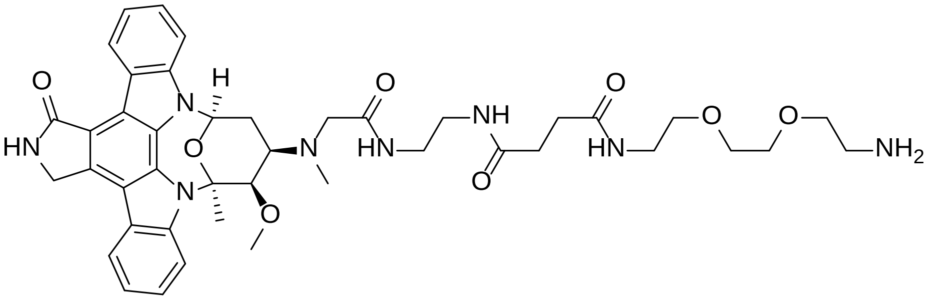

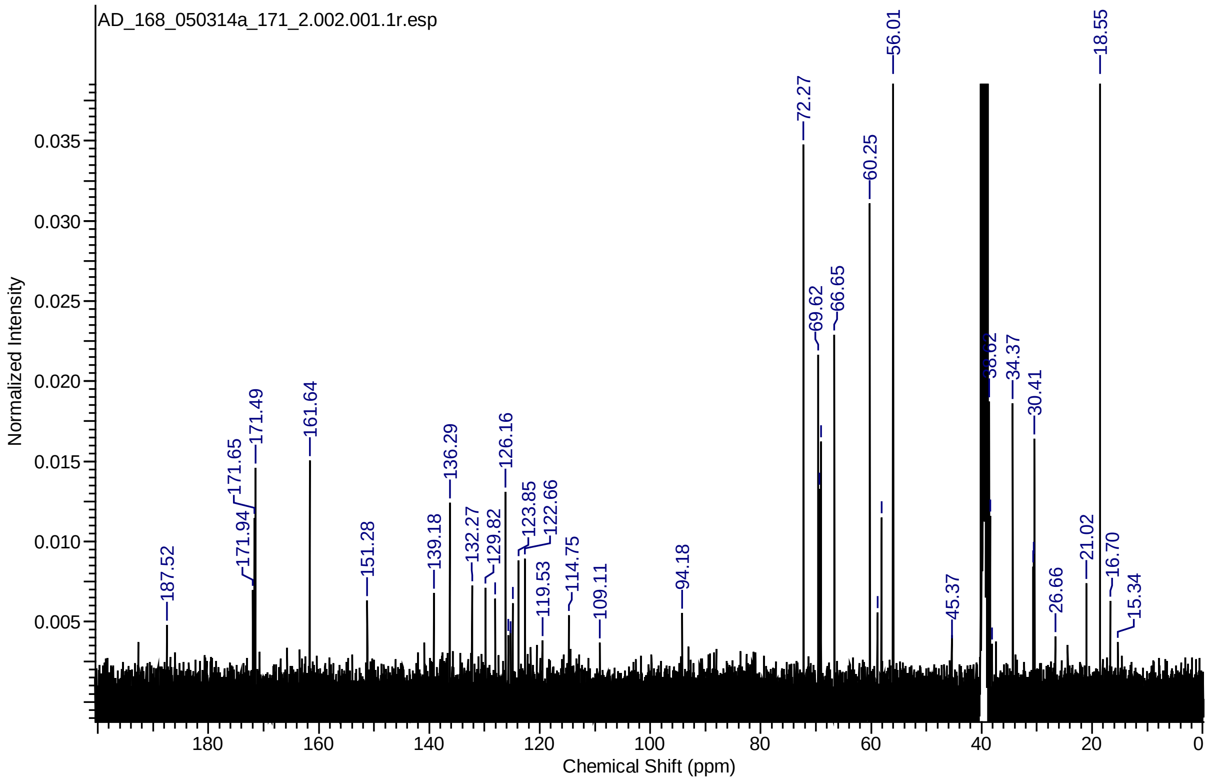

HPLC analysis of compound **7**

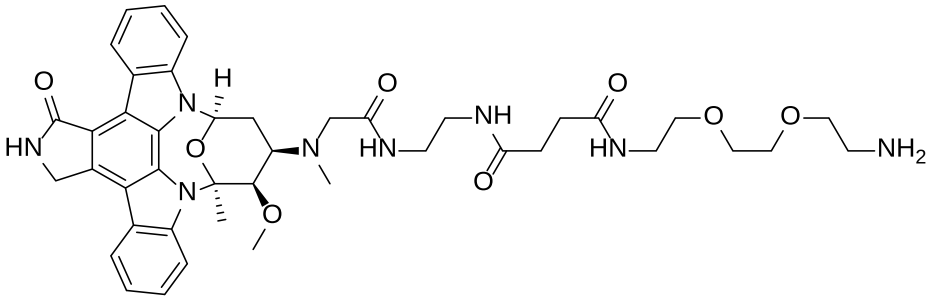

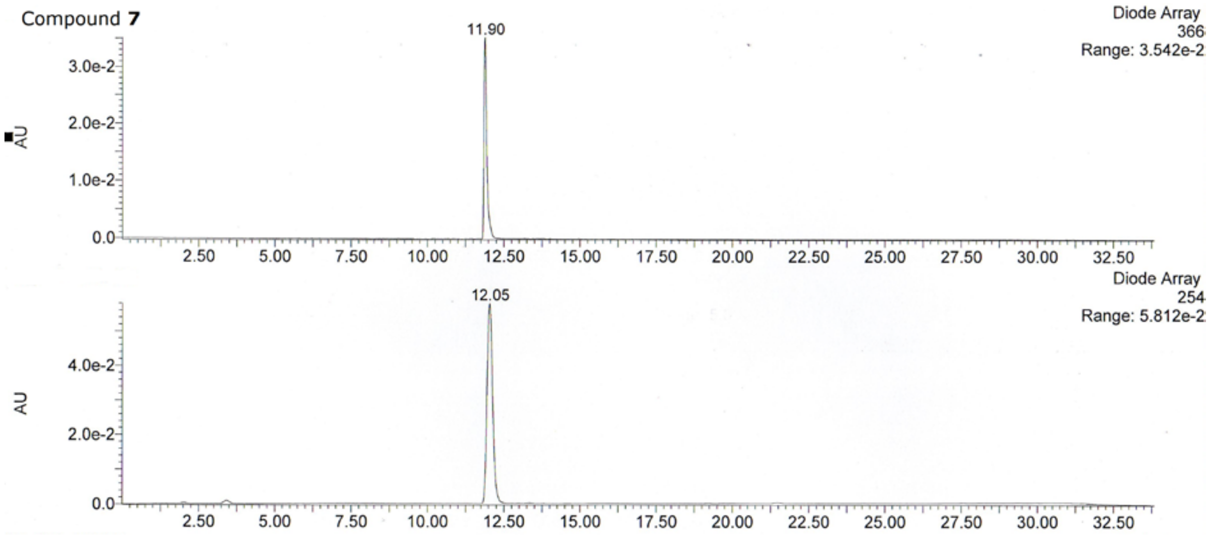

^{1}H spectrum for compound **8**

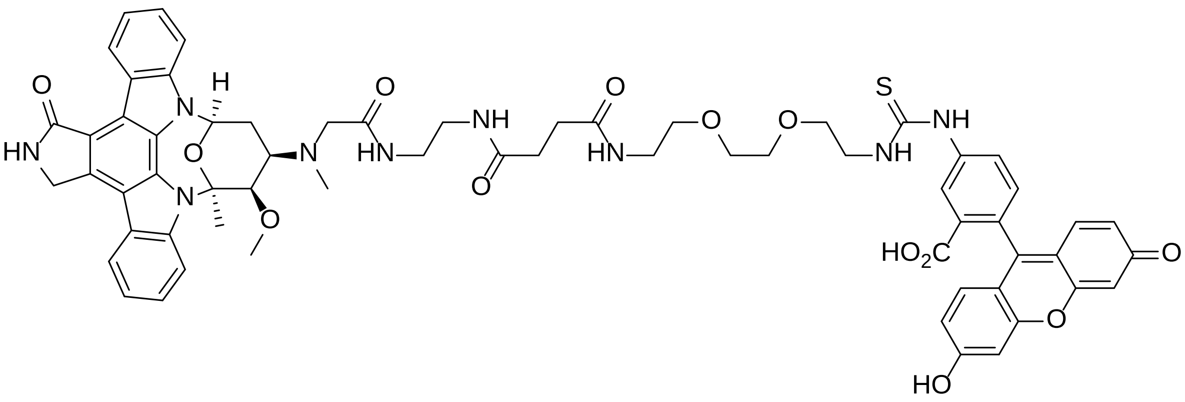

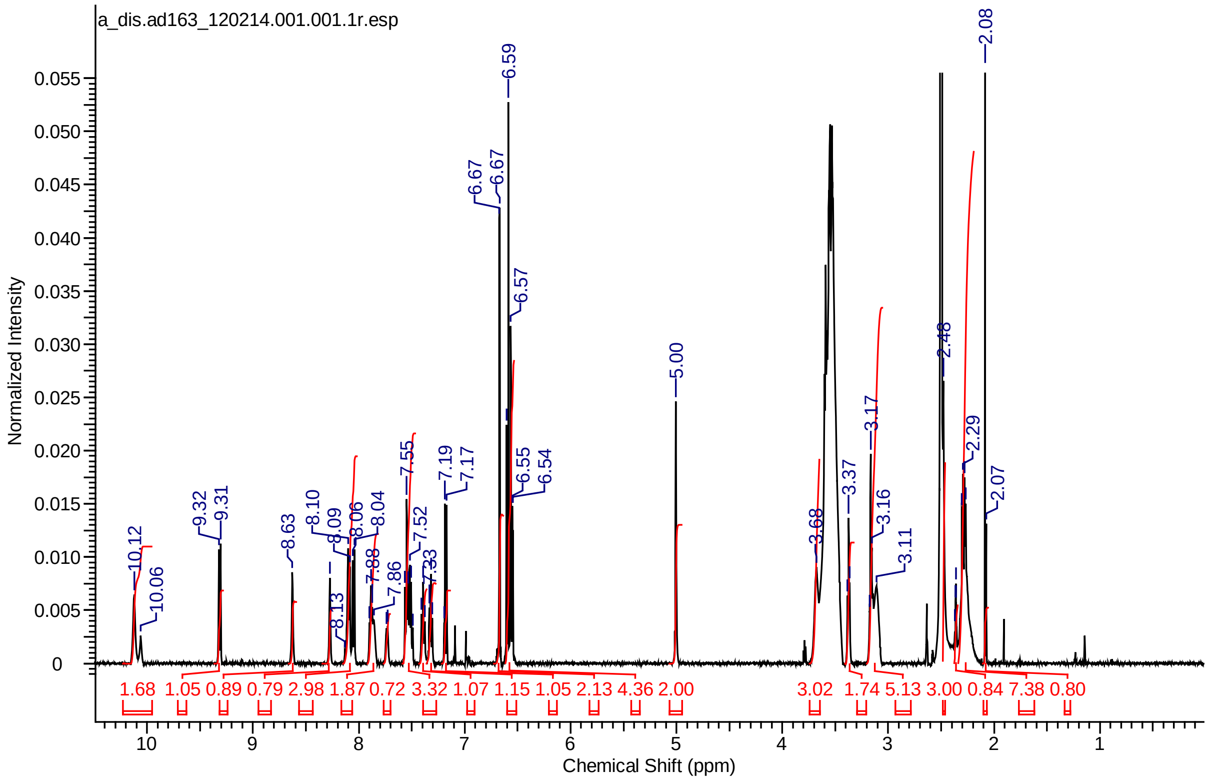

compound **8**

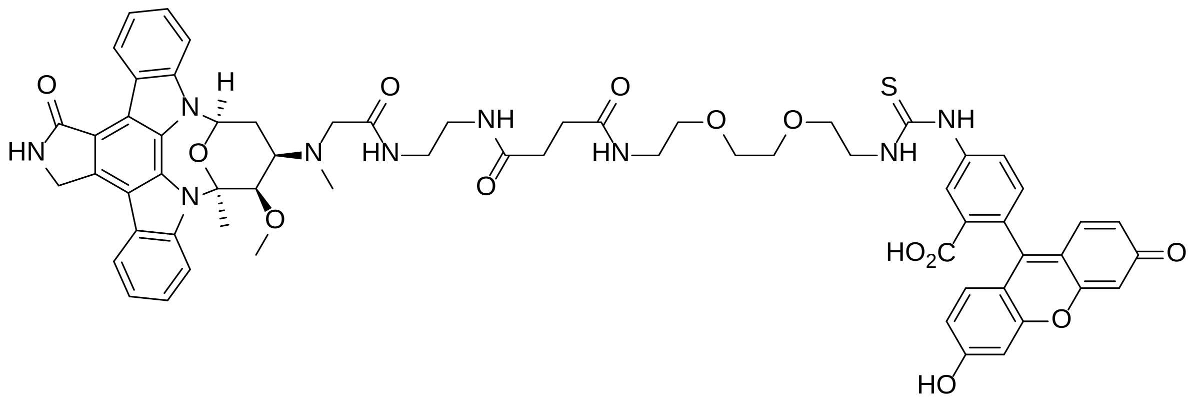

O
HN
N
H
O
N
O
N
O
HN
NH
O
O
HN
O
O
S
NH
NH
HO2C
O
O
HO

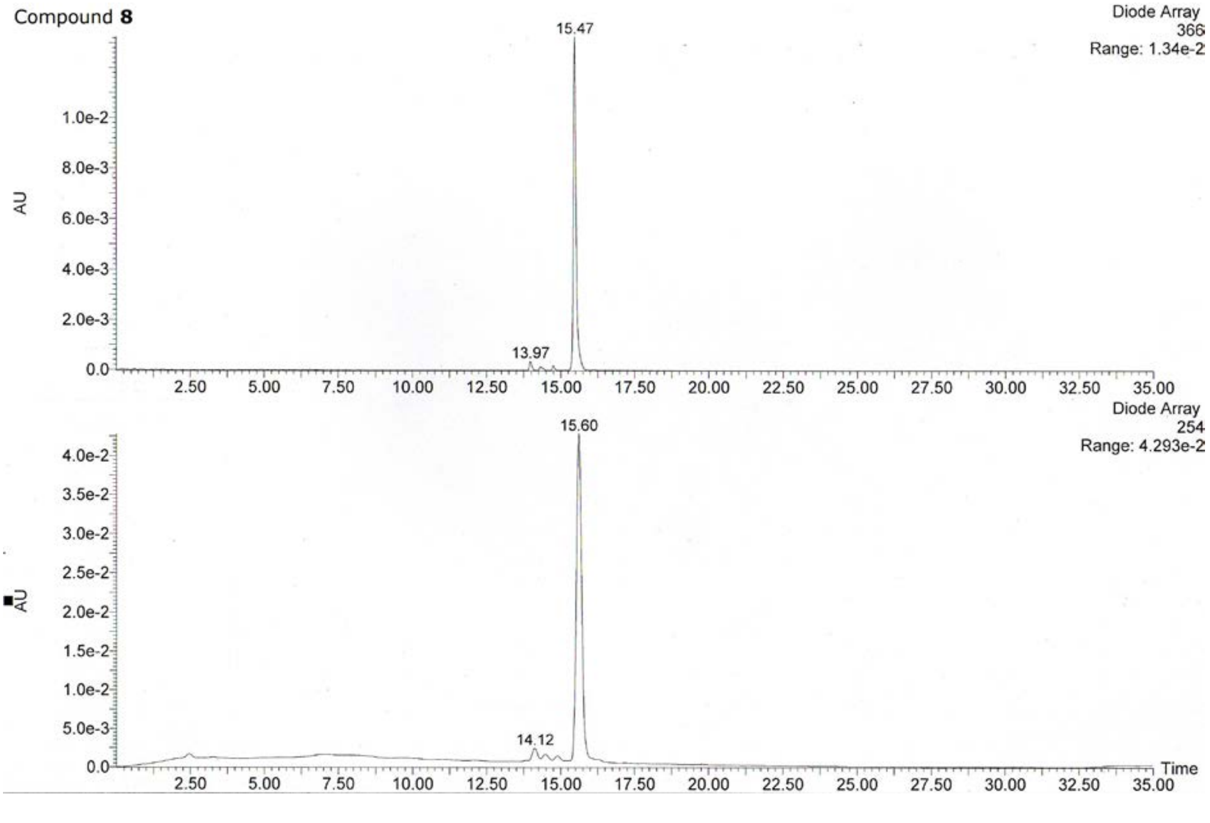

Compound 8
Diode Array
366
Range: 1.34e-2
15.47
13.97
AU
1.0e-2
8.0e-3
6.0e-3
4.0e-3
2.0e-3
0.0
2.50
5.00
7.50
10.00
12.50
15.00
17.50
20.00
22.50
25.00
27.50
30.00
32.50
35.00
Diode Array
254
Range: 4.293e-2
15.60
14.12
4.0e-2
3.5e-2
3.0e-2
2.5e-2
2.0e-2
1.5e-2
1.0e-2
5.0e-3
Time

Variable temperature spectra for compound **2**

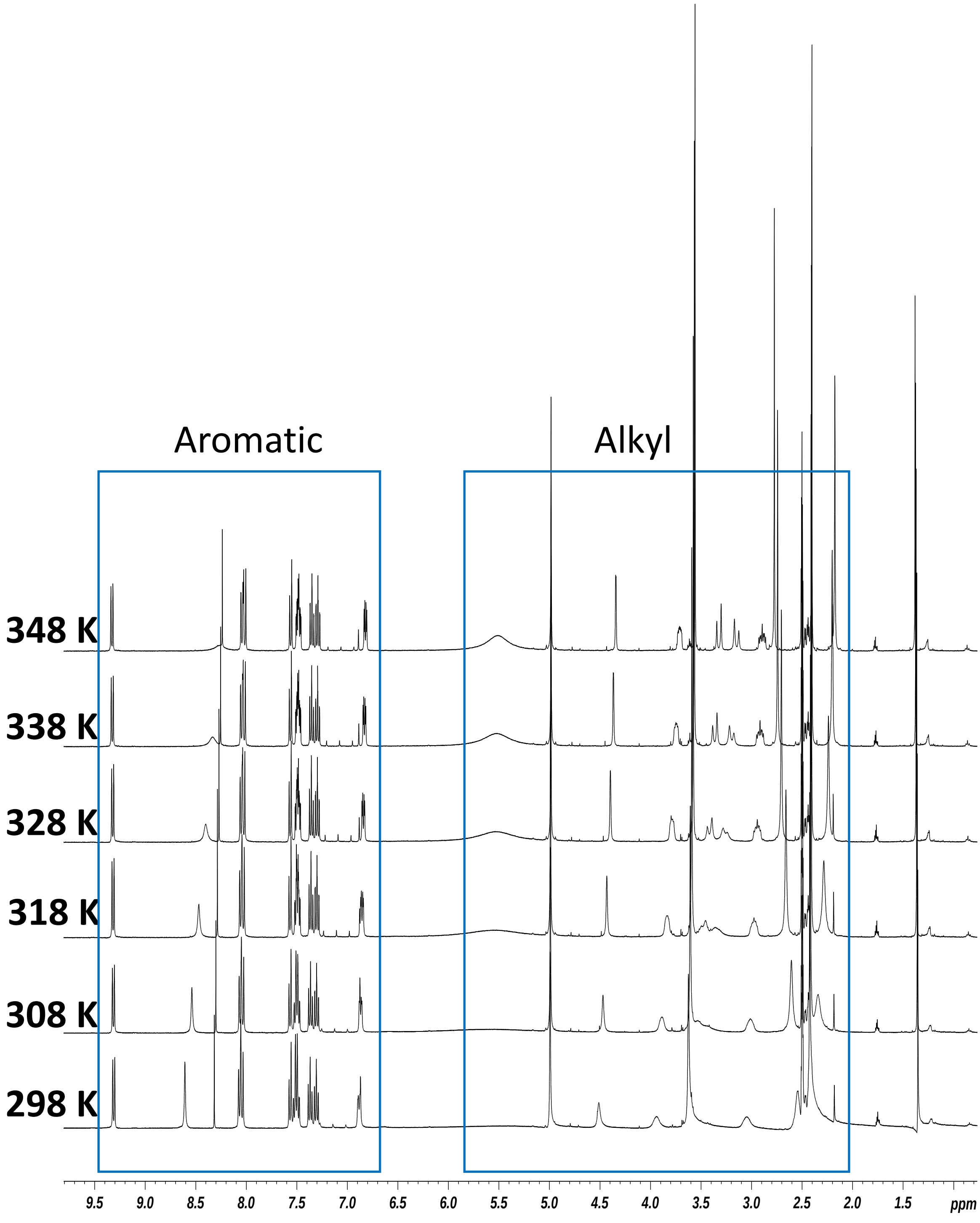

VT NMR experiment performed on **2** illustrating the lack of finely resolved features at room temperature (298K) due to rotameric effects. The spectra illustrate improved incremental signal resolution when the sample is heated from 298 to 348 K in 10 K increments.

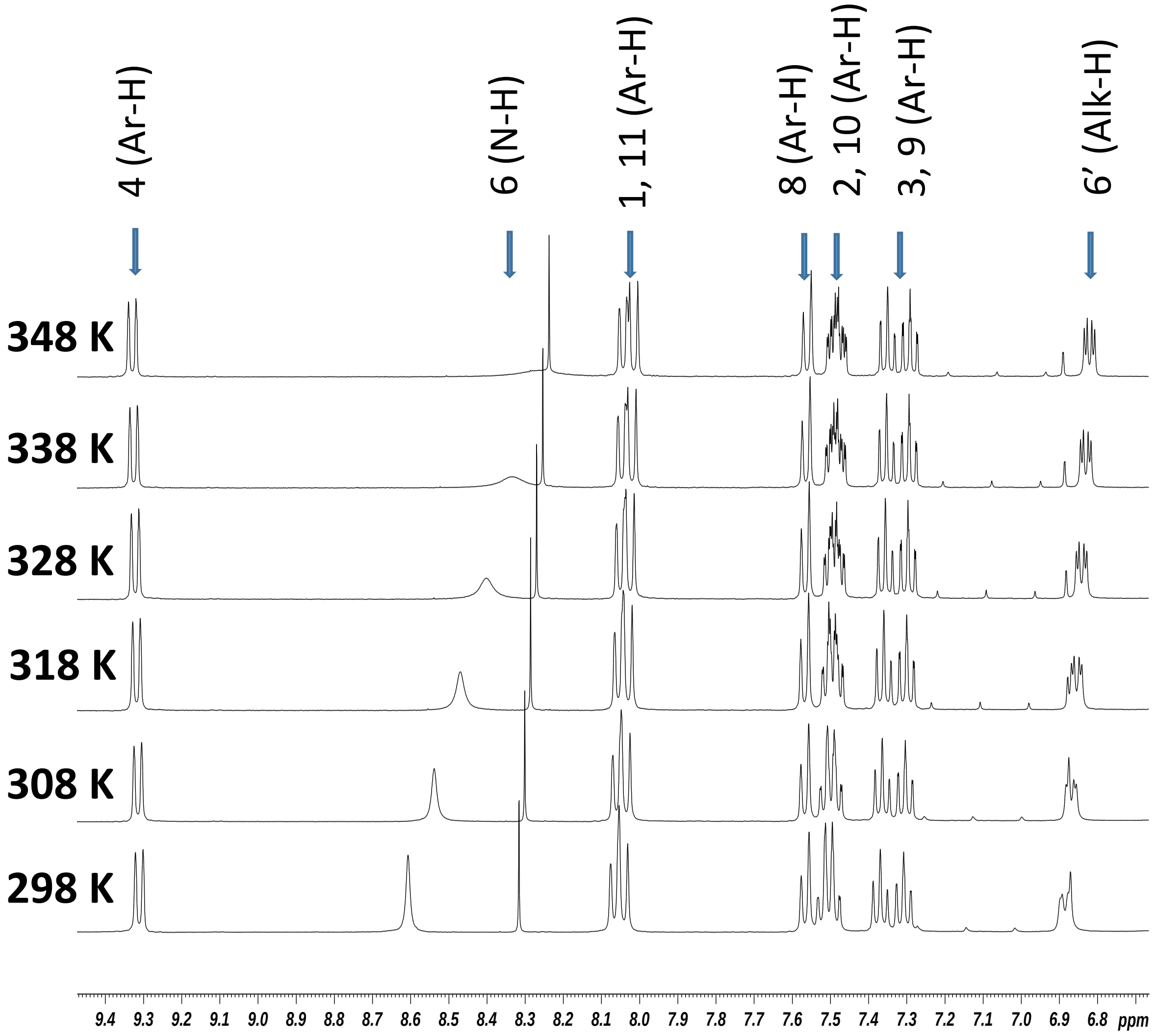

Enlargement of the low-field region of the VT NMR experiment on **2**. Assignments are given of which some remain constant with increasing temperature (*e.g.* the 4-position aromatic proton). Other signals show improved resolution, *e.g.* the 6' anomeric proton or a change in chemical shift, *e.g.* the 6 position amide -NH.

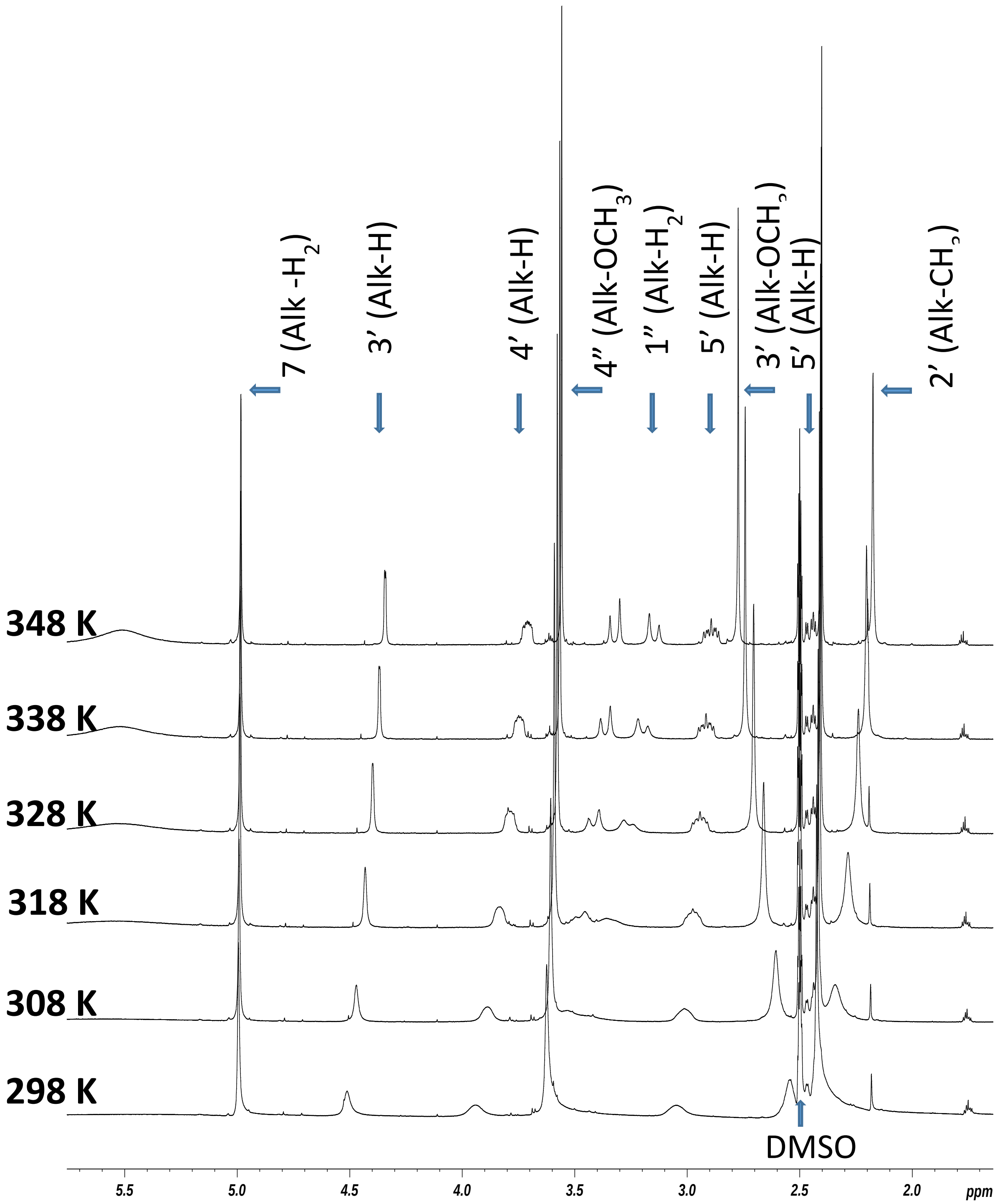

Enlargement of the high-field region of the VT NMR experiment on **2**. Small and broad singlets from the tetrahydropyran ring protons are observed at room temperature (298 K) while at 75 °C (348 K) fine detail is revealed.
